# Loss of the adaptor protein ShcA in endothelial cells protects against monocyte macrophage adhesion, LDL-oxydation, and atherosclerotic lesion formation

**DOI:** 10.1038/s41598-018-22819-3

**Published:** 2018-03-14

**Authors:** Antoine Abou-Jaoude, Lise Badiqué, Mohamed Mlih, Sara Awan, Sunning Guo, Alexandre Lemle, Clauda Abboud, Sophie Foppolo, Lionel Host, Jérôme Terrand, Hélène Justiniano, Joachim Herz, Rachel L. Matz, Philippe Boucher

**Affiliations:** 1 CNRS, UMR 7213, University of Strasbourg, 67401 Illkirch, France; 2 Department of Molecular Genetics, University of Texas Southwestern Medical Center, Dallas, TX USA

## Abstract

ShcA is an adaptor protein that binds to the cytoplasmic tail of receptor tyrosine kinases and of the Low Density Lipoprotein-related receptor 1 (LRP1), a trans-membrane receptor that protects against atherosclerosis. Here, we examined the role of endothelial ShcA in atherosclerotic lesion formation. We found that atherosclerosis progression was markedly attenuated in mice deleted for ShcA in endothelial cells, that macrophage content was reduced at the sites of lesions, and that adhesion molecules such as the intercellular adhesion molecule-1 (ICAM-1) were severely reduced. Our data indicate that transcriptional regulation of ShcA by the zinc-finger E-box-binding homeobox 1 (ZEB1) and the Hippo pathway effector YAP, promotes ICAM-1 expression independently of p-NF-κB, the primary driver of adhesion molecules expressions. In addition, ShcA suppresses endothelial Akt and nitric oxide synthase (eNOS) expressions. Thus, through down regulation of eNOS and ZEB1-mediated ICAM-1 up regulation, endothelial ShcA promotes monocyte-macrophage adhesion and atherosclerotic lesion formation. Reducing ShcA expression in endothelial cells may represent an obvious therapeutic approach to prevent atherosclerosis.

## Introduction

Atherosclerosis involves multiple processes such as endothelial dysfunction, inflammation and cell proliferation. It coincides with subendothelial low-density lipoprotein (LDL) accumulation. The pro-oxidative environment favors oxidation of LDL and oxidized LDL (oxLDL) activate endothelial cells which overexpress adhesion molecules E-selectin, VCAM-1 and ICAM-1^1^. Thus, activation of these signaling pathways in endothelial cells is a key mechanism in the development of atherosclerotic lesions, and controlling endothelial dysfunction could reduce the progression of the disease.

ShcA is a cytosolic adaptor protein^2^ that binds to the cytoplasmic tail of receptor tyrosine kinases (RTKs). Germ line deletion of the ShcA gene in mice leads to lethality at embryonic day 12, demonstrating an essential, but still undefined, role during development^3^. In adults and in embryos, ShcA regulates several important physiological processes. For instance, it signals in pathways such as IGF-I or PDGFβ, which are involved in proliferation/differentiation decisions^2,4–6^. These signals converge to Ras/MAP kinase and Akt/mTOR pathways. ShcA also binds to the tyrosine-phosphorylated form of the second NPxY motif within the tail of Low-density lipoprotein (LDL) receptor–Related Protein-1 (LRP1), an ubiquitously expressed transmembrane receptor that belongs to the LDL receptor gene family^7^. LRP1 is involved in lipoproteins endocytosis and in the control of intracellular signaling pathways. Mice lacking LRP1 in vascular smooth muscle cells (vSMCs) are characterized by a susceptibility to develop atherosclerosis. The lesions are associated with increased PDGFβ and TGFβ signaling that activate vSMCs proliferation^8^, and decreased Wnt5a signaling that stimulates foam cell formation^9,10^. The PDGF receptor and LRP1 co-immunoprecipitate and LRP1 is a substrate for PDGF-dependent tyrosine kinases^8,11,12^. Thus, by binding to LRP1, ShcA might play an important role in atherosclerotic lesions development.

ShcA is expressed in the cardiovascular system early during embryogenesis and in adults, and controls heart development^3^. In the heart, by binding to integrins or dystrophin, it links the extracellular matrix (ECM) to the cytoskeleton and the contractile apparatus^2,13^. In the vascular wall, the role of ShcA is not well defined. The mammalian ShcA protein has 3 isoforms of 46, 52 and 66 kDa and previous studies showed that mice lacking the p66 isoform had reduced tissue oxidative stress, foam cell and early atherosclerotic lesion formation when fed a high fat diet^14^. However, the molecular and cellular mechanisms of this phenotype remain largely unknown. In particular, it does not indicate in which vascular cell type ShcA deletion would be atheroprotective. We previously reported that the deletion of ShcA in vSMCs did not modify the development of atherosclerotic lesions in mice fed an atherogenic diet^13^. Here, to study the role of ShcA in atherosclerosis and vascular remodeling, we suppressed its expression specifically in endothelial cells using the Cre/lox system.

## Results

### Specific deletion of ShcA in endothelial cells protects from atherosclerosis

**Figure 1:**
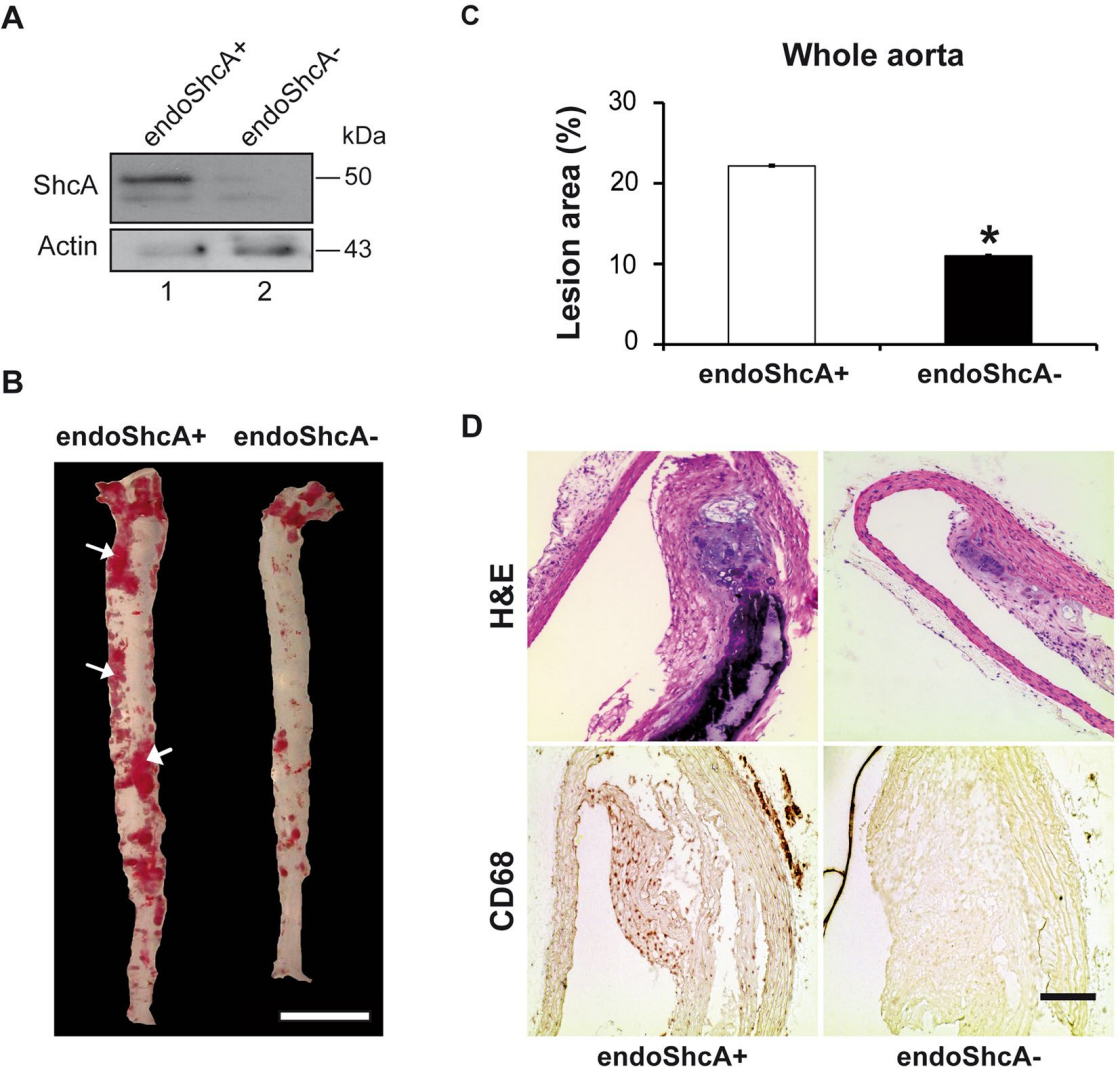
Absence of ShcA in endothelial cells protects from atherosclerotic lesion formation. Atherosclerosis in mice lacking ShcA in vascular endothelial cells and fed a cholesterol-rich diet. Western blotting of ShcA in endothelial cells isolated and pooled from aortas of endoShcA− and endoShcA+ mice (n = 5 mice for each genotype) **(A)**. Opened and Sudan IV-stained aortas from endoShcA− mice and controls (endoShcA+). Arrows show lipid-laden (Sudan-positive) atherosclerotic lesions; Scale bar, 0.5 cm **(B)**. Quantification of atherosclerotic lesion size in whole aortas from endoShcA− (*n = *5) and control (*n = *5) mice **(C)**. Hematoxylin and eosin (H&E) and CD68 staining of the lesions in thoracic aortas from endoShcA− and endoShcA+ mice. Scale bar, 20 μm **(D)**. Data are represented as mean ± SEM. *P < 0.05, two-tailed unpaired Student’s t-test.

We generated Tie2Cre+/ShcA^flox/flox^ mice, in which ShcA is selectively ablated in endothelial cells, by inter-crossing Tie2Cre transgenic mice with floxed ShcA animals^13^ (ShcA^flox/flox^). To increase atherosclerosis susceptibility, Tie2Cre+/ShcA^flox/flox^ animals were maintained on a LDL receptor-deficient background (LDLR−), fed an atherogenic diet, and are hereafter referred to as endoShcA-. Western blot analysis of ShcA in endothelial cells isolated from aortas of endoShcA- and endoShcA+ (control) mice confirmed the deletion of ShcA (Fig. 1A). Absence of ShcA expression in endothelial cells had no significant effect on plasma cholesterol (48.3 ± 10.1 mmol/l in endoShcA− mice vs 41.2 ± 3.3 mmol/l in controls) or triglyceride levels (2.5 ± 0.1 mmol/l in endoShcA− mice vs 2.9 ± 0.9 mmol/l in controls), in mice fed an atherogenic diet for 24 weeks. However, when fed an atherogenic diet atherosclerotic lesions were two times smaller in endoShcA− mice than in age-matched control mice (endoShcA+) as demonstrated by Soudan IV staining and *en face* analysis of the whole aortas (Fig. 1B,C), and histological analysis (Fig. 1D, top panels). The reduced atherosclerotic lesion size was similar in male and female mice (data not shown). During atherosclerosis, infiltration of foamy macrophages and vascular smooth muscle cells plays a crucial role. Whereas histological analysis of the arterial wall in large vessels such as thoracic aortas revealed an accumulation of CD-68-positive macrophage foam cells within the core of the atherosclerotic plaques in control mice (Fig. 1D, bottom panels), almost no CD68-positive macrophage-foam cells accumulated in endoShcA− aortas (Fig. 1D, bottom panels). This decreased number of macrophage foam cells in the atherosclerotic plaques indicates that ShcA expression in endothelial cells promotes CD-68-positive macrophage cell infiltration and/or foam cell formation.

### Deletion of ShcA in endothelial cells protects from intracellular lipid accumulation and foam cell formation

**Figure 2:**
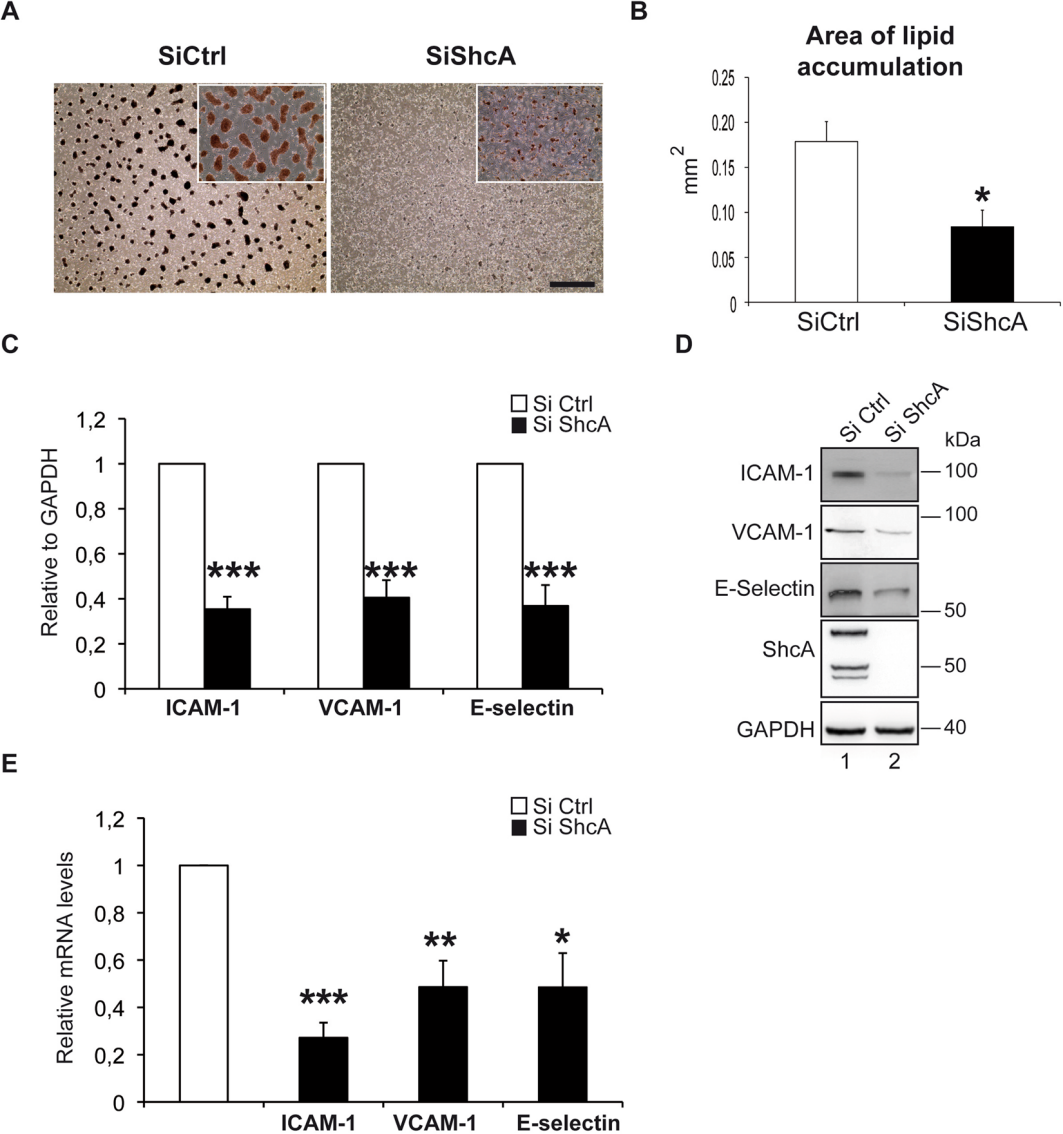
Expression of the three isoforms of ShcA in endothelial cells is required for foam cells formation, macrophage adhesion, and ICAM-1 expression. Accumulation of lipids in THP1-derived macrophages treated with oxidized LDL in the absence or presence of the three isoforms of ShcA in EA.hy 926 endothelial cells. Cells were stained with Oil/RedO. Representative microscopic fields. The subpanels on the right are higher magnification (2.5×) images. SiCtrl and SiShcA panels are same magnifications, and scale bare is 5 µm (**A**). Quantification of lipid accumulation upon Oil/RedO staining in THP1-derived macrophages treated with oxidized LDL in the absence (n = 5) or presence (n = 5) of ShcA in EA.hy 926 endothelial cells. Oil/RedO positive regions were manually outlined and the quantification of outlined regions was determined with Image J as described in the methods section (**B**). Quantification by western blot of indicated genes in EA.hy 926 endothelial cells down regulated for ShcA (siShcA) (n = 3) and in control cells (siCtrl) (n = 3) (**C**). Western blot analysis of ICAM-1, VCAM-1, E-Selectin, ShcA, and GAPDH expressions in EA.hy 926 endothelial cells down regulated for ShcA (siShcA) and in control cells (siCtrl) (**D**). mRNA of the indicated genes measured by Real Time PCR in EA.hy 926 endothelial cells down regulated for ShcA (siShcA) (n = 3) and in control cells (n = 3) transfected with siControl (siCtrl) (**E**). All data are represented as mean ± SEM. *P < 0.05, **P < 0.01, ***P < 0.001, two-tailed unpaired Student’s t-test.

To study the role of endothelial ShcA on intracellular lipid accumulation and foam cell formation, human endothelial cells (EA.hy 926 cell line) down regulated for p66, p52 and p46 ShcA isoforms and control cells were co-cultured in presence of THP-1 monocyte-derived macrophages stimulated with oxidized low-density lipoprotein to induce foam cell formation. Deletion of three isoforms of ShcA in endothelial cells significantly decreases the ox LDL uptake of macrophages and foam cell formation as evidenced by Oil-Red-O staining (Fig. 2A). Quantification analysis upon Oil-Red-O staining showed half neutral lipid accumulation in the absence of endothelial ShcA (Fig. 2B). These data indicate that endothelial ShcA promotes lipid accumulation in macrophages and foam cell formation.

### Decrease of ICAM-1 expression in the absence of ShcA

Recruitment of monocytes and their endothelial cell adhesion occurs through intercellular adhesion molecule 1 (ICAM-1), vascular cell adhesion molecule 1 (VCAM-1), and E-selectin, secreted by inflamed or damaged endothelium. Among these, the key molecule ICAM-1, a member of the adhesion immunoglobulin super family^15^, displays an important role in the development of atherosclerosis. For instance, deficiency in ICAM-1 was shown to disable monocyte–endothelial cell adhesion leading to reduce atherosclerotic lesion size in apoE−/− mice^16^. To test whether decreased accumulation of CD-68-positive foam cells was due to decreased expression of ICAM-1 in endothelial cells, we measured its mRNA and protein levels in EA.hy 926 endothelial cells down regulated for p66, p52 and p46 ShcA isoforms and in control cells. We found a marked decrease of ICAM-1 protein (Fig. 2C,D) and mRNA levels (Fig. 2E) in the absence of ShcA. VCAM-1 mRNA, and E-selectin mRNA and protein levels were also decreased (Fig. 2C–E).

### Deletion of the p66 isoform of ShcA in endothelial cells is not sufficient to protect from intracellular lipid accumulation and foam cell formation

**Figure 3:**
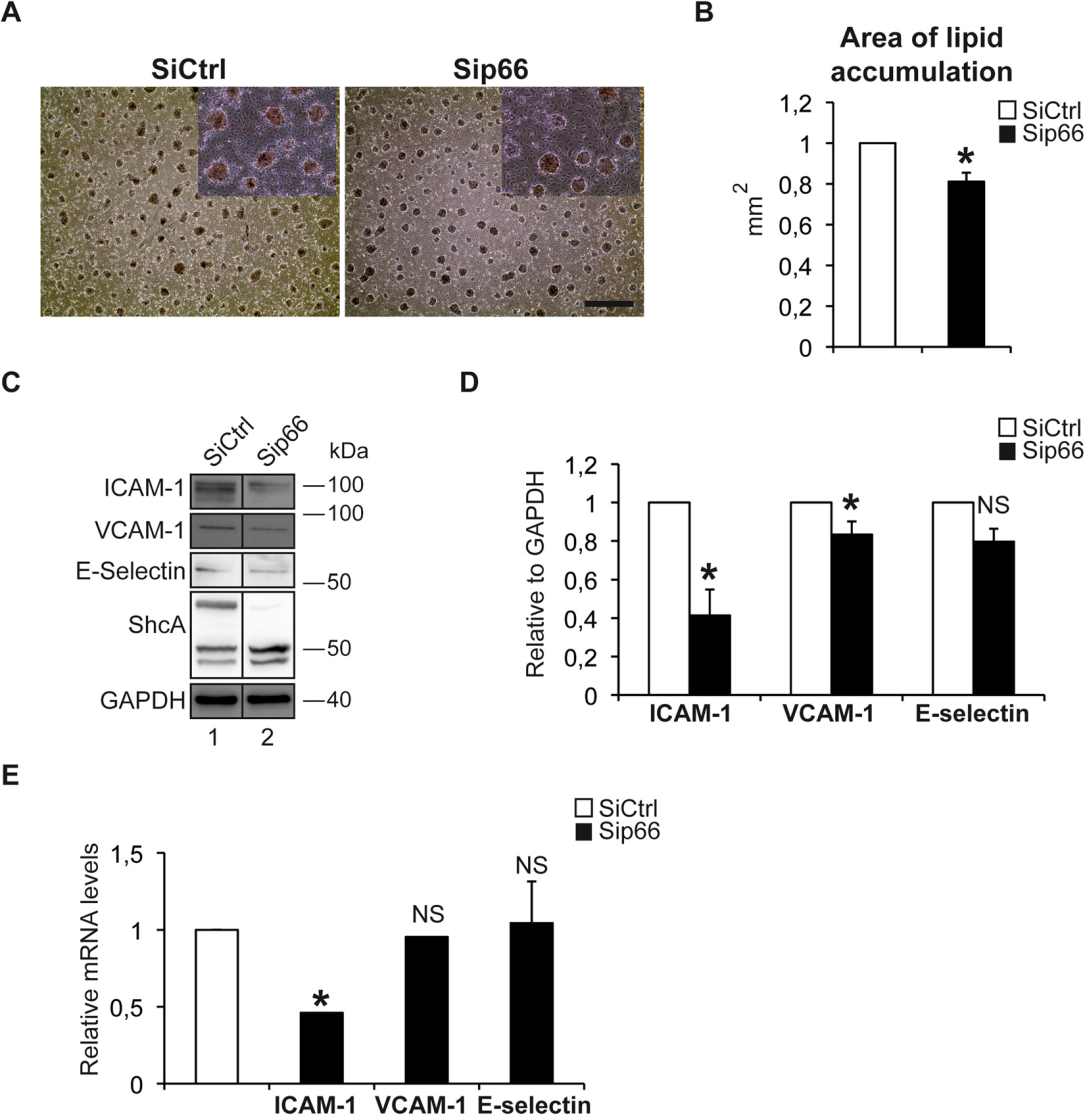
Deletion of p66ShcA down regulates ICAM-1 expression in endothelial cells, but does not decrease ox LDL uptake of THP-1 monocyte-derived macrophages and foam cell formation. Accumulation of lipids in THP1-derived macrophages treated with oxidized LDL in the absence or presence of p66ShcA in EA.hy 926 endothelial cells. Cells were stained with Oil/RedO. Representative microscopic fields. The subpanels on the right are higher magnification (2.5×) images. SiCtrl and p66SiShcA panels are same magnifications, and scale bare is 5 µm (**A**). Quantification of lipid accumulation upon Oil/RedO staining in THP1-derived macrophages treated with oxidized LDL in the absence (n = 5) or presence (n = 5) of p66ShcA in EA.hy 926 endothelial cells. Oil/RedO positive regions were manually outlined and the quantification of outlined regions was determined with Image J as described in the methods section (**B**). Western blot analysis (**C**) and quantification of western blot analysis of ICAM-1 in EA.hy 926 endothelial cells down regulated for p66ShcA and in controls (**D**). RT–PCR of the indicated genes in EA.hy 926 endothelial cells down regulated for p66ShcA and in controls (**E**). Error bars, s.e.m. *P < 0.05, NS = non significant.

Since genetic deletion of the p66 isoform of ShcA reduces oxLDLuptake and early atherosclerotic plaque formation in apolipoprotein E-/- fed a high fat diet^14,17^, we next wanted to test whether deletion of the p66 isoform in endothelial cells is sufficient to protect from intracellular lipid accumulation and foam cell formation. When EA.hy 926 endothelial cells down regulated for the p66 isoform of ShcA were co-cultured in presence of THP-1 monocyte-derived macrophages stimulated with oxidized low-density lipoprotein to induce foam cell formation, we found a modest decrease in foam cell formation as evidenced by Oil-Red-O staining (Fig. 3A). Quantification analysis showed a 20% decrease in neutral lipid accumulation in the absence of p66 ShcA (Fig. 3B). These results suggest that three isoforms of ShcA are required for efficient intracellular lipid accumulation and foam cell formation. We also tested whether deletion of the p66 ShcA isoform decreased expression of adhesion molecules. We found a marked decrease of ICAM-1 protein (Fig. 3C,D) and mRNA levels (Fig. 3E) in EA.hy 926 endothelial cells down regulated for the p66 isoform of ShcA compared to control cells. However, VCAM-1 protein levels were only moderately decreased, whereas VCAM-1 mRNA as well as E-selectin mRNA and protein levels remained unchanged (Fig. 3D,E). Thus, in endothelial cells p46, p52 and p66 ShcA play an important role in expression of adhesion molecules and in the interaction between endothelial cells and monocytes, leading to monocyte recruitment and subsequent development of atherosclerosis.

### Increase in p-NF-κB expression in ShcA−/− endothelial cells

**Figure 4:**
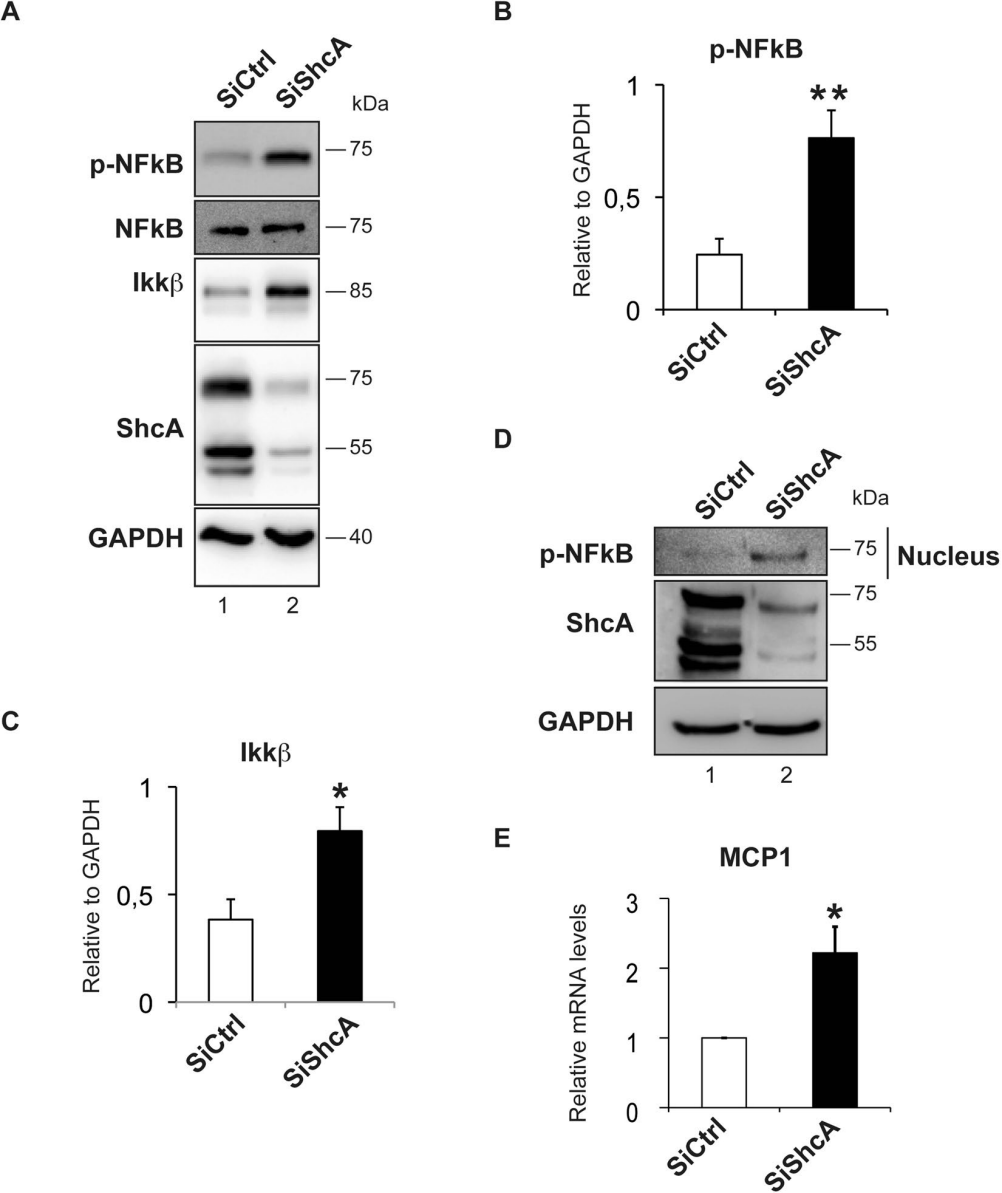
ShcA in endothelial cells triggers ICAM-1 expressions independently of NF-kB. Western blot analysis of the p65 subunit of NF-kB phosphorylated at the Ser536 residue (p-NF-kB), total NFkB p65 (NF-kB), IKKβ, ShcA and GAPDH in whole cell lysate from EA.hy 926 endothelial cells down regulated for the three isoforms of ShcA (siShcA) and in controls cells (siCtrl) **(A)**. Quantification by western blot of the p65 subunit of p-NF-kB protein levels (*n = *6) **(B)** and of IKKβ protein levels (*n = *7) in whole cell lysate **(C)**. Western blot measurement of p-NFkB nuclear accumulation in EA.hy 926 endothelial cells down regulated for ShcA (siShcA) compared to controls cells (siCtrl) (representative from n = 3 separated experiments) **(D)**. The increase in nuclear translocation of p-NFkB in EA.hy 926 endothelial cells down regulated for ShcA is accompanied by an increase in its target gene mRNA levels, MCP1 (n = 5 for each genotype) **(E)**. All data are represented as mean ± SEM. *P < 0.05, **P < 0.01, two-tailed unpaired Student’s t-test.

We next wanted to determine how ShcA regulates ICAM-1 expression in endothelial cells. The nuclear form of the NF-kappa B transcription factor (NF-κB) binds to DNA as a heterodimer of a 50 kDa (p50) and 65 kDa (p65) polypeptide^18^. Once phosphorylated, the transcription factor NF-κB is the major driver of *VCAM-1*, *ICAM-1* and *E-selectin* expression^18^. Surprisingly, treatment of endothelial cells with siRNA against ShcA triggered the phosphorylation of the p65 subunit of NF-κB (Fig. 4A,B) and increases the expression of its endogenous activator IKKβ (Fig. 4A,C)^19^. To determine whether NF-κB is activated in the absence of ShcA, we measured its nuclear translocation and activation of its target gene MCP1^20,21^. We found that in EA.hy 926 endothelial cells down regulated for ShcA (siShcA), p-NF-κB accumulated in the nucleus (Fig. 4D) and activated MCP1 (Fig. 4E) when compared to control cells (siCtrl). This suggests that, in the absence of ShcA regulation of adhesion molecules expression levels is independent of NF-κB.

### Decrease in ShcA expression activates nuclear translocation of the transcription factor ZEB1 and decreases the nuclear translocation of YAP

**Figure 5:**
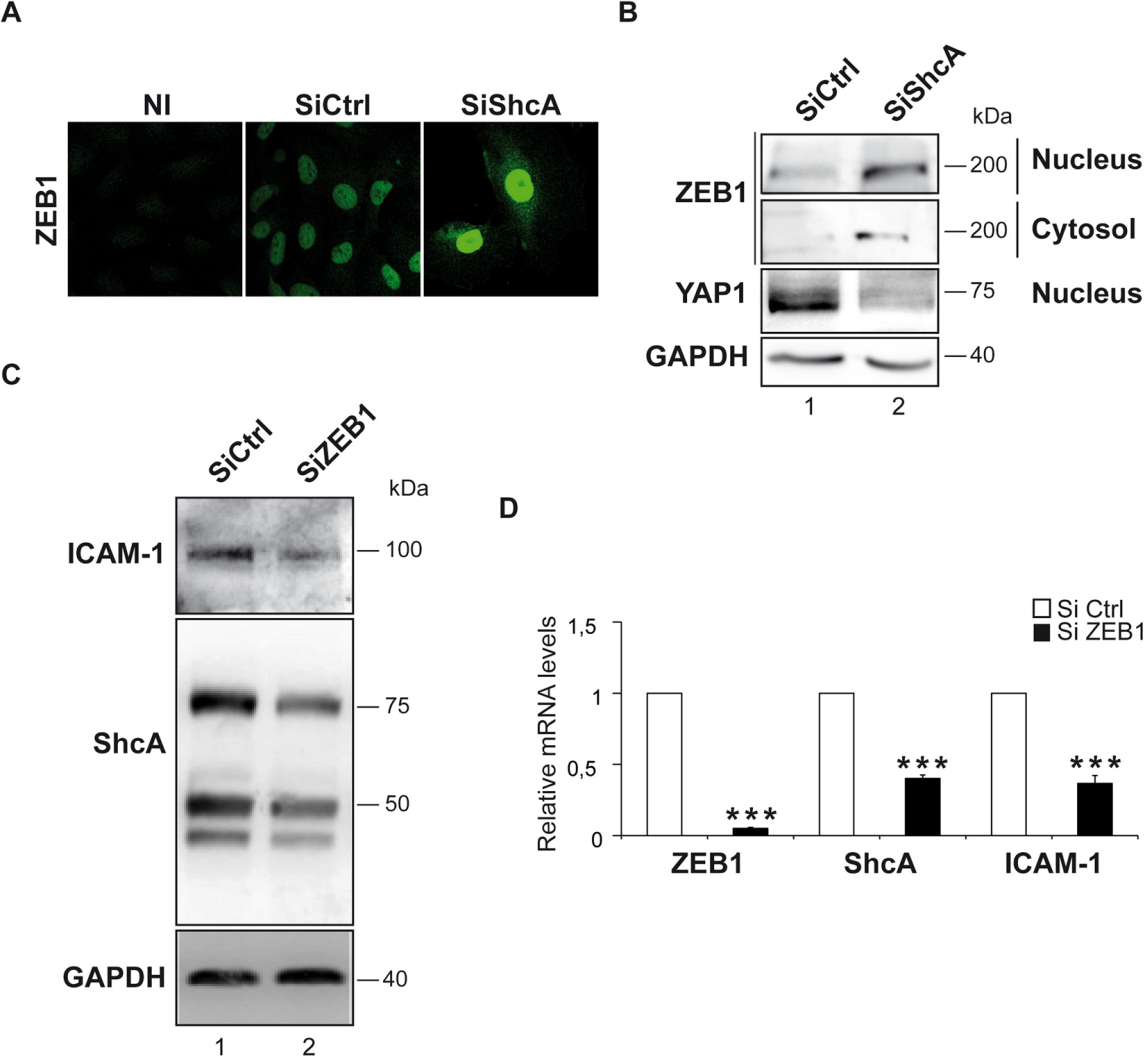
The absence of ShcA increased ZEB1 nuclear translocation and decreased YAP nuclear translocation. To follow accumulation of ZEB1 in the nucleus, EA.hy 926 endothelial cells down regulated for the three isoforms of ShcA (siShcA) and control cells (siCtrl) were labeled with anti-ZEB1 and analyzed by confocal images **(A)**. Western blot analysis of the indicated genes in cytosol and nuclear fractions from EA.hy 926 endothelial cells down regulated for the three isoforms of ShcA (siShcA) and control cells (siCtrl) (*n = *4) **(B)**. Western blot analysis of ShcA and ICAM1 in whole cell lysates from EA.hy 926 endothelial cells down regulated for ZEB1 (siZEB1) (n = 3) and control cells (n = 3) (siCtrl) **(C)**. ZEB1, ShcA and ICAM1 mRNA levels analyzed by Real Time PCR in EA.hy 926 endothelial cells down regulated for ZEB1 (siZEB1) (n = 3) and control cells (n = 3) (siCtrl) **(D)**. NI, non-immun antibody. Data are represented as mean ± SEM. ***P < 0.001, two-tailed unpaired Student’s t-test.

It was previously reported that the transcription factor zinc-finger E-box-binding homeobox 1 (ZEB1) bound the ZEB1-binding sites of ShcA promoter in epithelial cells^22^. In addition, ZEB1 has at least one putative binding site on the p46/p52 isoforms promoter at the position −830 (Eukaryote promoter and GP miner databases). Thus, we next thought to assess the nuclear translocation of ZEB1 in EA.hy 926 endothelial cells treated with siShcA and in control cells. Using immuno-fluorescence (Fig. 5A) and cell fractionation experiments (Fig. 5B), we found, a marked increase in cytosolic expression and nuclear translocation of ZEB1 in siShcA-treated endothelial cells *versus* control cells indicating that ShcA is required for down regulation of ZEB1 expression. To evaluate the impact of reducing ZEB1 levels on ShcA and ICAM-1 expressions, we measured ShcA and ICAM-1 mRNA levels upon siZEB1 treatment. Interestingly, down regulation of ZEB1 decreased both ShcA and ICAM-1 mRNA and protein levels (Fig. 5C–D). Knockdown of ZEB1 similarly decreased p66 ShcA mRNA levels (data not shown). These data indicate that ShcA and ZEB1 are required for ICAM-1 transcriptional regulation, whereas ShcA down regulates ZEB1 expression through a negative feedback mechanism. Mechanistically, ZEB1 function as a transcriptional repressor^23^. However, depending upon the recruitment of a different set of co-factors, direct transcriptional activation by ZEB1 has also been reported for a few target genes^24,25^. For instance, ZEB1 directly binds to YAP to stimulate transcription^24^. Since ShcA is required for nuclear translocation of YAP^26^, ShcA might recruit YAP in the nucleus to switch ZEB1 from a repressor to a transcriptional activator, thereby up-regulating ICAM-1 expression. To test this, we performed cell fractionation assays in EA.hy 926 endothelial cells and found a decrease in nuclear expression of YAP in ShcA knockdown cells compared to controls (Fig. 5B). This strongly suggests that a ZEB1/YAP complex, most likely through binding to ShcA promoters, promotes ShcA-mediated up regulation of ICAM-1 expression.

### Increased p-Akt that activates eNOS, decreases LDL oxidation and recruitment of monocytes

**Figure 6:**
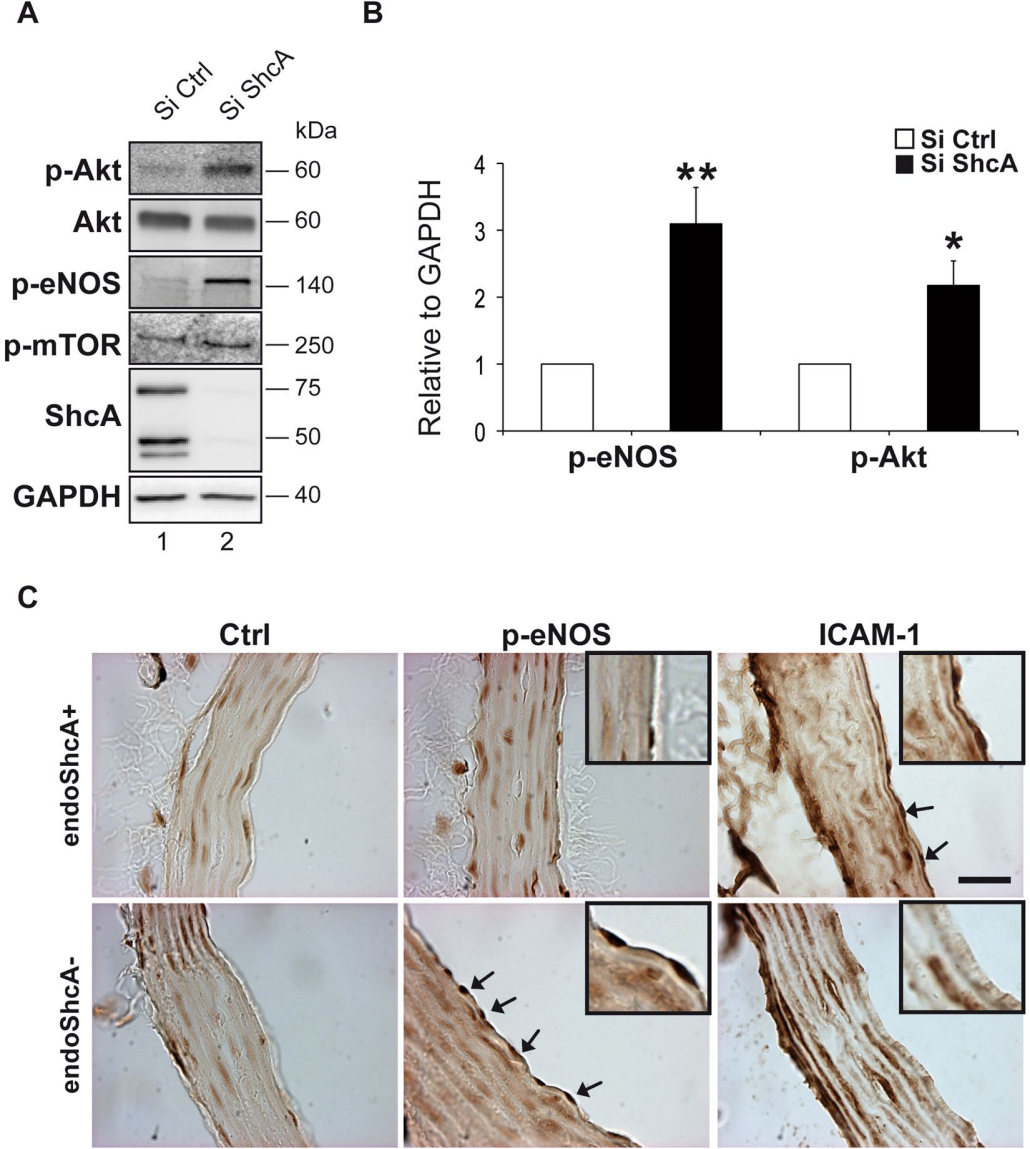
The decrease in ICAM-1 protein expression correlates with increased p-Akt and p-eNOS protein levels in endothelial cells deficient for ShcA. Western blot analysis of whole cell lysates from EA.hy 926 endothelial cells down regulated for the three isoforms of ShcA and in control cells using using phospho-eNOS (Ser1177) (p-eNOS), phospho-Akt (Ser473) (p-Akt), phospho-mTOR (Ser2448) (p-mTOR), Akt, ShcA, and GAPDH antibodies (**A**). Quantification of western blot analysis of p-eNOS and p-Akt in EA.hy 926 endothelial cells down regulated for ShcA and in controls (*n = *8 separated experiments) (**B**). Representative immunohistochemistry experiment for expression of p-eNOS and ICAM-1 in mice lacking ShcA in vascular endothelial cells (endoShcA−) (n = 2) or control mice (endoShcA+) (n = 2) fed a cholesterol-rich diet as described in the method section. Decreased ShcA expression in endothelial cells of mice (endoShcA−) greatly increased expression of p-eNOS protein (arrows), as well as decreased expression of ICAM-1 (arrows) in these cells, compared to controls (endoShcA+). Non-immune antibodies (Ctrl), the subpanels on the right are higher magnification (2.5×) images of vascular endothelial cells. Scale bar, 20 μm (**C**). Data are represented as mean ± SEM. *P < 0.05, **P < 0.01, two-tailed unpaired Student’s t-test.

In endothelial cells, Akt can phosphorylate and activate endothelial nitric oxide synthase (eNOS) which inhibits LDL oxidation^27^. As loss of eNOS activity is an established contributor to endothelial dysfunction^28^ and endothelium-derived NO plays a vital role in the prevention of atherosclerosis^29^, we explored whether absence of ShcA increased eNOS activation. In EA.hy 926 endothelial cells down regulated for ShcA we found an increase in p-Akt protein levels (Fig. 6A,B) and its target gene p-mTOR (Fig. 6A)^30^, accompanied by a marked increased in eNOS phosphorylation (Fig. 6A,B). Densitometric analysis confirmed these data (Fig. 5B). Interestingly, immunohistochemistry analysis show that mice lacking ShcA in vascular endothelial cells (endoShcA−) and fed a cholesterol-rich diet expressed a larger amount of p-eNOS than control mice (Fig. 6C). Furthermore, they also expressed less ICAM-1 in vascular endothelial cells than controls (Fig. 6C). Thus, through inhibition of eNOS, increased expression of ICAM-1, and increased LDL oxidation, and by promoting maladaptive monocyte-derived macrophage adhesion, endothelial ShcA contributes to atherosclerotic lesion formation.

## Discussion

Our data indicate that three isoforms of endothelial ShcA protein play a pivotal role in the pathophysiology of atherosclerosis by triggering monocyte-derived macrophages infiltration and LDL oxidation. Previous genetic ablation studies suggested an important role for ShcA in the regulation of fat accumulation^31^. Other studies reported that constitutive mutation of p66Shc gene, one of the three isoforms encoded by the mammalian ShcA locus, decreased atherosclerotic lesion size in mice fed a high-fat diet^14,32^. Interestingly, lesions from p66 (Shc−/−) mice had less macrophage-derived foam cells than wild type mice, and decreased proatherogenic factors, including oxLDL, but, the mechanism was unknown. *In vitro*, p66 Shc is involved in oxLDL uptake^17^. Here, we report that all three isoforms of ShcA are required to decrease eNOS expression levels, to increase ICAM-1 expression, and promote cell adhesion molecules and atherosclerosis.

Our study also reveals a complex interaction between ShcA and ZEB1 that promotes adhesion molecules expression in EA.hy 926 endothelial cells. In absence of ShcA, ICAM-1 and other adhesion proteins are down regulated. Interestingly, we found that NF-κB, the primary driver of adhesion protein expression is upregulated in the absence of ShcA. This would suggest that ShcA upregulated adhesion protein expression through a mechanism independent of NF-κB. Since ZEB1 binds to the E-box consensus sequence CACCT present in the p66 promoter^22^, and that such sequences are also present on the p46/p52 isoforms promoter, we tested whether ZEB1 activated ShcA in EA.hy 926 endothelial cells. We found that upon siZEB1 treatments, ShcA and ICAM-1 are markedly down regulated indicating that ZEB1 induces ShcA expression. In addition, ShcA knockdown is accompanied by an increase in ZEB1 expression levels and its nuclear localization in endothelial cells. This would represent an adaptive response to ShcA loss and a negative feedback loop between ShcA and ZEB1 in endothelial cells. Such a negative feedback mechanism was already described in lung epithelial cancer cells where p66Shc deficiency enhanced the expression of ZEB1 and consequently decreased E-cadherin^22^.

ZEB1 is a transcriptional repressor of epithelial genes such as E-cadherin^23^. However, ZEB1 can also behave as an activator depending on its interaction with co-factors^24^. Indeed, by binding to the Hippo-pathway effector YAP (Yes-associated protein), ZEB1 switches from a repressor to an activator of transcription^24^. Here we found that in the absence of ShcA, YAP protein expression decreased in the nucleus. This strongly suggests that ShcA participate in the dual function of ZEB1. By promoting YAP nuclear translocation ShcA switches ZEB1 function from a repressor to an activator of transcription, thereby promoting ICAM-1 expression. However, we cannot exclude that interactions with other transcription factors may also confer the transcriptional co-activation by ZEB1.

Here, we also found that upon ShcA knockdown, the transcription factor NF-κB is induced and translocated to the nucleus. This would stimulate inflammation, recruitment of multiple immune cells including macrophages, and promote atherosclerosis. Since NF-κB increases ZEB1 nuclear translocation, which in turn behaves as a repressor of E-cadherin^33^, similarly, in the absence of ShcA, NF-KB might repress ICAM-1 expression through increased ZEB1 nuclear translocation. Thus, whereas NF-κB stimulates inflammation, the increase in NF-κB can be not only limited to negative effects on atherosclerosis.

Previous studies reported that LDL cholesterol upregulates the expression of human endothelial p66Shc via hypomethylation of CpG dinucleotides in the p66Shc promoter^34^, and that epigenetic upregulation of the p66Shc isoform of ShcA mediates increase in ICAM-1 expression^34^. This suggest that at least two independent mechanisms up regulate ShcA and the corresponding increase in ICAM-1 expression in endothelial cells, one involving hypomethylation of the promoter, another one involving transcriptional up regulation and nuclear translocation of ZEB1.

Endothelium-derived NO has also a crucial role in the local regulation of vascular homeostasis. A decrease in the bioavailability of NO aggravates the development of atherosclerotic lesions^35^. NO *per se* suppresses LDL oxidation and macrophage accumulation^36^. eNOS is an Akt target and the activation of eNOS by Akt is one of the most important physiological effects of Akt on cell attachment in endothelial cells^27^. Here we found that p-Akt is decreased when ShcA is expressed. This results by decreased eNOS levels, LDL oxidation, inflammation and atherosclerosis in mice.

In conclusion, we found that ShcA promotes a ZEB1-mediated increase of ICAM-1 expression, favor monocyte-derived macrophages adhesion, intracellular lipid accumulation and foam cell formation while simultaneously decreasing vascular NO production, events that would contribute to endothelial dysfunction commonly seen during atherosclerosis.

## Methods

### Mice

All animal experimentations and procedures were approved by the Institutional Animal Care and Use Committee (IACUC) of University of Strasbourg, France, and performed conform to the guidelines from Directive 2010/63/EU of the European Parliament on the protection of animals used for scientific purposes. C57/B6 mice carrying a ShcA allele into which loxP sites are integrated have been generated by gene targeting in embryonic stem cells. LoxP sites have been introduced upstream of exon 2 and downstream of exon 7 (ShcA^flox/flox^)^13^. Cre-mediated recombination resulted in deletion of a 2-kb fragment containing the sequence encoding the PTB domain required for binding to phosphorylated receptors and for signaling activity. Endothelial cells specific p66, p52 and p46 ShcA inactivation was achieved by crossing transgenic mice carrying the Tie2-Cre transgene (a kind gift from Masashi Yanagisawa, University of Texas Southwestern Medical Center, at Dallas) with ShcA^flox/flox^ mice. In order to increase susceptibility to spontaneous atherosclerotic lesion development, these animals were crossed to LDL receptor knock out mice (LDLR −/−). Genotyping of the wild type and ShcA mutant mice by polymerase chain reaction (PCR) was performed as described^37^ using primers specific for ShcA (Primers available upon request). Animals were maintained on a 12-h light/12-h dark cycle. For *in vivo* analysis, 4 males and 1 female of three to four months old were used for each genotype, fed a Paigen diet during six months, and analyzed for atherosclerotic lesions as described previously^8^. For the isolation of tissue for further analysis, the agents used for euthanasia were ketamine (750 mg/kg) and xylazine (50 mg/kg), intraperitoneally.

### Purification of lipoproteins

LDLs were isolated from South-American sourced Fetal Bovine serum by density gradient centrifugation. LDLs were dialyzed for 48 hours in a solution of NaCl 150 mM and 0.24 mM disodium EDTA, pH 7.4. LDLs were then centrifuged for 30 minutes, 4 °C at 10,000 RPM and filtered through a 0.22 µm filter. Concentrations were determined by the Lowry method.

### Oxidation of LDLs

LDLs were added at a concentration of 600 µg/ml in RPMI 1640 medium supplemented with 10% South-American sourced FBS. Copper sulfide (SIGMA) was dissolved in RPMI medium, filtered through a 0.22 µm filter and added to the LDL solution to a final concentration of 10 µM. The resulting solution was incubated at 37 °C for 48 hours.

### Cells

Aortic endothelial cells isolation: Thoracic and abdominal aortas were carefully dissected from 3 months old control and Tie2Cre+/ShcAflox/flox mice. The endothelial layer was removed immediately after dissection by intraluminal perfusion with 0.5% 3-[(3-cholamidopropyl)dimethylammonio]-1 propane sulphonate (CHAPS) in Physiological salt solution (PSS) for 20 s followed by repeated washing with PSS. The washed endothelial layers from 5 mice were collected, pooled and submitted to SDS-polyacrylamide gel electrophoresis and immunoblot analysis according to standard procedures.

Human EA.hy 926 endothelial cells (a kind gift from Dr Sophie Martin, UMR CNRS 7213, University of Strasbourg) were seeded in 6-well-plates at 120.000 cells/well in DMEM medium supplemented with 10% FBS and 2 mM L-Glutamine, and were placed in a 37 °C incubator with humidified atmosphere containing 5% CO2 as described^38,39^. 24 hours after seeding, cells were transfected with either scrambled siRNA, siRNA against p66, p52 and p46 isoforms of ShcA (Dharmacon) or siRNA against the p66 isoform of ShcA^40^ (Dharmacon, custom siRNA, 5′-GAAUGAGUCUCUGUCAUCGUU-3′) at a final concentration of 100 nM using lipofectamine 3000. 48 hours post-transfection, DMEM medium was replaced with the LDL oxidation solution containing THP-1 (a kind gift from Prof Florence Toti, UMR CNRS 7213, University of Strasbourg) Macrophage cells at 2.10^6^ cells/well. Phorbol myristate acetate (PMA) was added at a concentration of 50 ng/ml to induce macrophage differentiation. Cells were then incubated at 37 °C, 5%CO_2_ for 48 hours.

Control EA.hy 926 endothelial cells transfected with scrambled siRNAs or siShcA were incubated in the same conditions but in a medium that did not contain oxidized LDLs. At the same time, 2.10^6^ cells/well THP-1 cells were incubated in the same conditions for 48 hours and used as positive control. 48 hours later, wells were stained using Oil/RedO staining. We controlled SiRNA transfection efficiency using Western Blotting and/or Real Time PCR. Each experiment was repeated 5 times for statistical significance. For quantitative analysis, images taken through the microscope were processed using ImageJ. Regions formed by the accumulation of foam cells were manually outlined and the quantification of outlined region was determined by ImageJ.

### Lipid Staining

The cells were fixed with 10% Paraformaldehyde for 30 minutes, washed twice with phosphate-buffered saline (PBS) buffer, pH = 7.4, and stained with a saturated concentration of oil red O for a minimum of 30 minutes. Cells were then washed twice with PBS buffer and LDL accumulation by THP1 cells was observed under microscopy; representative images of whole wells were taken for further analysis.

### Gene expression analysis

RNA was isolated using TRIzol reagent (Sigma, St Louis, Mo) according to the manufacturer’s instructions. 50 ng of RNA were converted to cDNA using the High-capacity cDNA Archive kit (Applied Biosystems, Foster City, CA). PCR amplification was performed using SYBRGreen PCR master mix (Kappa biosystems, Wilmington, MA) according to the manufacturer’s instructions. Primers sequences are available upon request.

### Histology experiments

For immunostaining and histology experiments, mice were transcardially perfused with a 4% paraformaldehyde solution in phosphate buffered saline. Entire aortas were fixed with 4% paraformaldehyde in phosphate-buffered saline, embedded in paraffin, and cut in 5 µm slices as described^37^. Sections were stained with hematoxylin and eosin as described^37^.

### Western blot

Whole cell extracts were fractionated by SDS-PAGE and transferred to a nitrocellulose membrane using a transfer apparatus according to the manufacturer’s protocols (Bio-Rad). After incubation with 5% BSA TBST (10 mM Tris, pH 8.0, 150 mM NaCl, 0.5% Tween 20) for 60 min at room temperature, the membrane was incubated at 4 °C overnight with primary antibodies directed against ShcA (Upstate), ICAM-1 (Abcam), VCAM-1 (Abcam), CD62E (E-selectin) (Abcam), NFkB p65 (Cell Signaling Technology), p-NFkB p65 (S536) (Cell Signaling Technology), Ikkbeta (Cell Signaling Technology), p-eNOS (S1177) (Cell Signaling Technology), P-mTOR (S2448) (Cell Signaling Technology), AREB6 (ZEB1) (Abcam), YAP1 (Santa Cruz), p-Akt (S473) (Cell Signaling Technology), Akt (Cell Signaling Technology), Actin (Sigma, St Louis, Mo) or GAPDH (Sigma, St Louis, Mo). Membranes were then washed five times for 5 min and incubated with a 1:10000 dilution of horseradish peroxidase-conjugated anti-mouse or anti-rabbit antibodies (Santa Cruz) for 1 h at room temperature. Blots were washed with TBST five times and developed with the ECL system according to the manufacturer’s protocols (BIO RAD Clarity™ Western ECL Substrate). Clarity™ western ECL substrate allowed visualization of protein expression using ImageQuant™ LAS 4000 Imaging System (Amersham). Optical densitometry was performed with Adobe PhotoshopCS and Image J normalizing bands intensity for GAPDH.

### Cell fractionation

For cell fractionation, monolayers of human endothelial cells (EAhy) were transfected with either scramble siRNA or siRNA against ShcA (Dharmacon) (100 nM). 48 hours post-transfection, cells were then fractionated as previously described^41^.

### Confocal Microscopy

EAhy cells were seeded on glass slides, 24 hours later, they were treated with either scramble siRNA or siRNA against ShcA. 48 hours post-transfection, the cells were fixed with paraformaldehyde, and incubated with anti-ZEB1 or anti-IgG control primary antibodies and Alexa Fluor 488 secondary antibodies. Immunofluorescence-labeled cells were analyzed using a Leica TSC SPE confocal microscope with the ×63 oil immersion objective.

### Statistical analysis

Values are reported as mean ± SEM of at least triplicate determinations. Statistical significance (*P* < 0.05) was determined using an unpaired Student’s *t* test (GraphPad Prism, *Abacus Concepts, Berkeley, CA*). P-values < 0.05, < 0.01, and < 0.001 are identified with 1, 2, 3 asterisks, respectively. ns: p > 0.05.

## Electronic supplementary material

Supplementary Information - Full blots
