# Author Correction: Loss of the adaptor protein ShcA in endothelial cells protects against monocyte macrophage adhesion, LDL-oxydation, and atherosclerotic lesion formation

**DOI:** 10.1038/s41598-018-27564-1

**Published:** 2018-06-19

**Authors:** Antoine Abou-Jaoude, Lise Badiqué, Mohamed Mlih, Sara Awan, Sunning Guo, Alexandre Lemle, Clauda Abboud, Sophie Foppolo, Lionel Host, Jérôme Terrand, Hélène Justiniano, Joachim Herz, Rachel L. Matz, Philippe Boucher

**Affiliations:** 1 CNRS, UMR 7213, University of Strasbourg, 67401 Illkirch, France; 2 Department of Molecular Genetics, University of Texas Southwestern Medical Center, Dallas, TX USA

Correction to: *Scientific Reports* 10.1038/s41598-018-22819-3, published online 14 March 2018

The original version of this Article contained a typographical error in the spelling of the name of author Antoine Abou-Jaoude, which was incorrectly given as Antoine Abou Jaoude. This has now been corrected in the PDF and HTML versions of the Article, and in the accompanying Supplementary Information file.

