# Supplementary material for: Loss of the adaptor protein ShcA in endothelial cells protects against monocyte macrophage adhesion, LDL-oxydation, and atherosclerotic lesion formation
Source: Sci Rep. 2018 Mar 14;8:4501. doi: 10.1038/s41598-018-22819-3 (PMC5852050; doi:10.1038/s41598-018-22819-3)
Supplement: Supplementary file 1 — Supplementary Information - Full blots [file 41598_2018_22819_MOESM1_ESM.pdf]

Loss of the adaptor protein ShcA in endothelial cells protects against monocyte macrophage adhesion, LDL-oxydation, and atherosclerotic lesion formation

Antoine Abou-Jaoude[1], Lise Badiqué[1], Mohamed Mlih[1], Sara Awan[1], Sunning Guo[1], Alexandre Lemle[1], Clauda Abboud[1], Sophie Foppolo[1], Lionel Host[1], JérômeTerrand[1], Hélène Justiniano[1], Joachim Herz[2], Rachel L. Matz[1]and Philippe Boucher[1]

1 CNRS, UMR 7213, University of Strasbourg, 67401 Illkirch, France

2 Department of Molecular Genetics, University of Texas Southwestern Medical Center, Dallas

Figure 1A

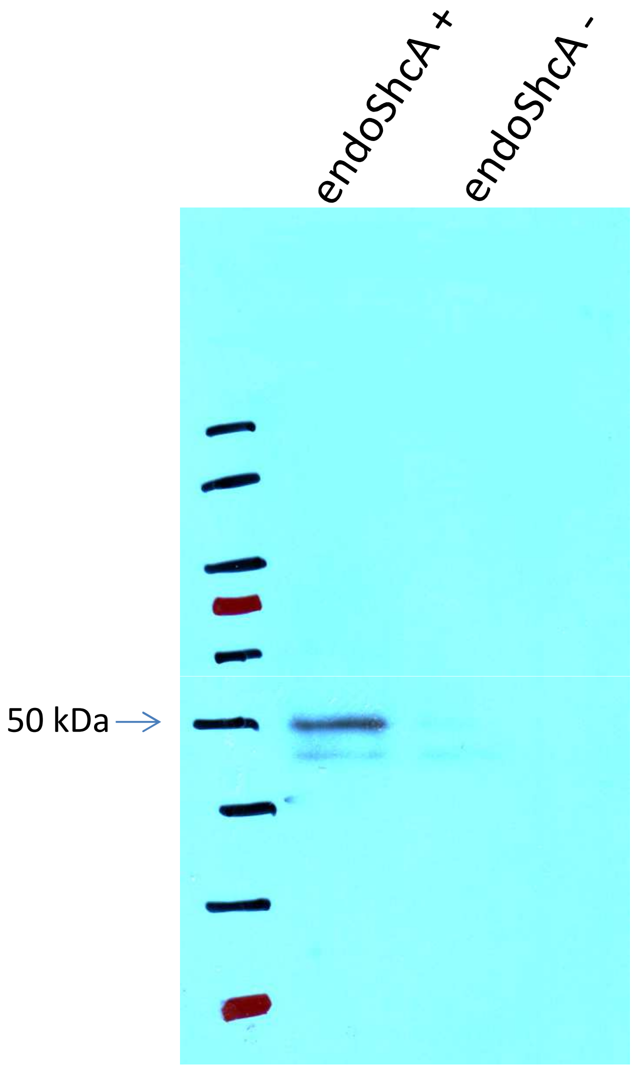

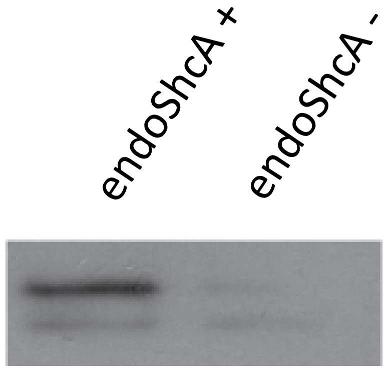

ShcA
66, 52, 46 kDa

Figure 1A

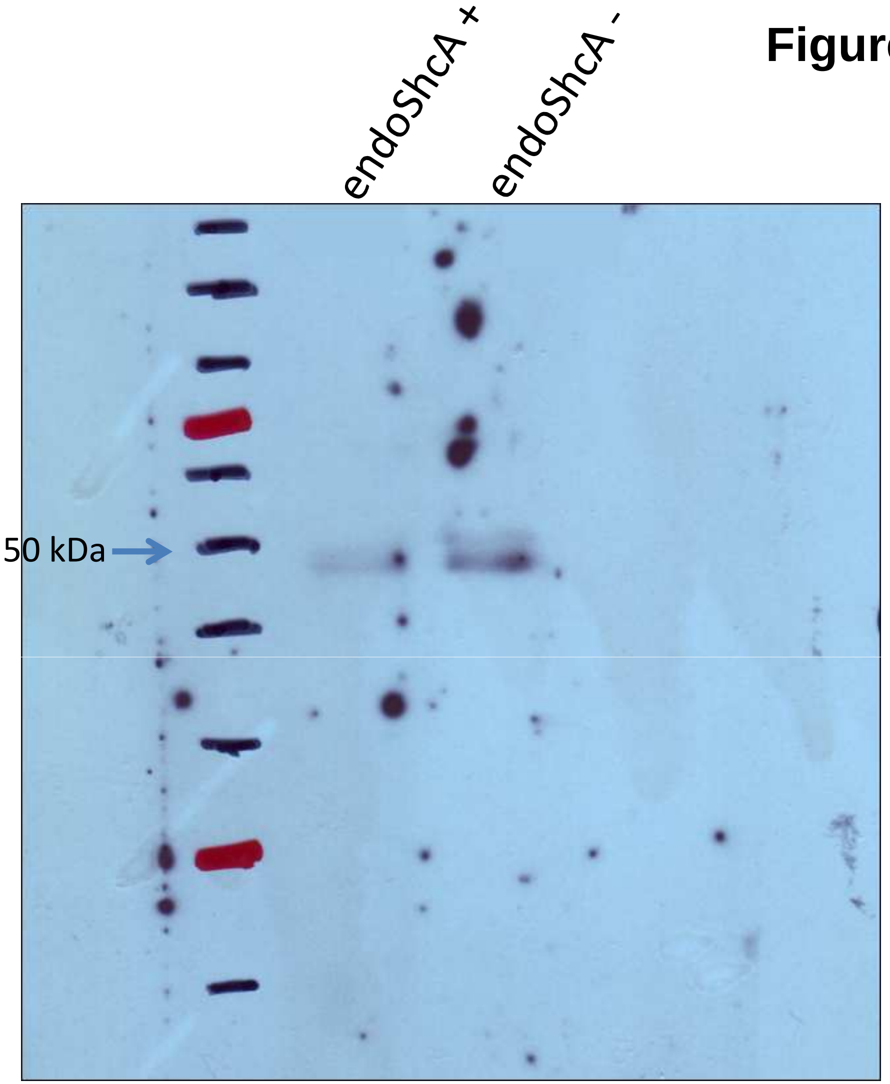

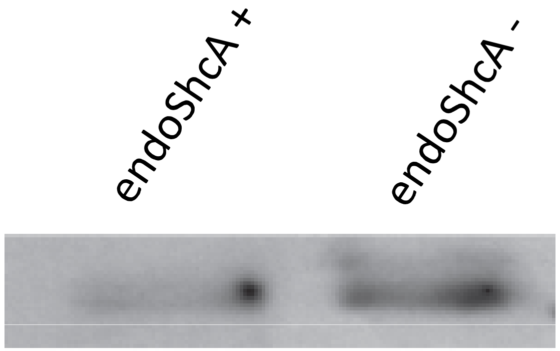

Actine
43 kDa

Figure 2D

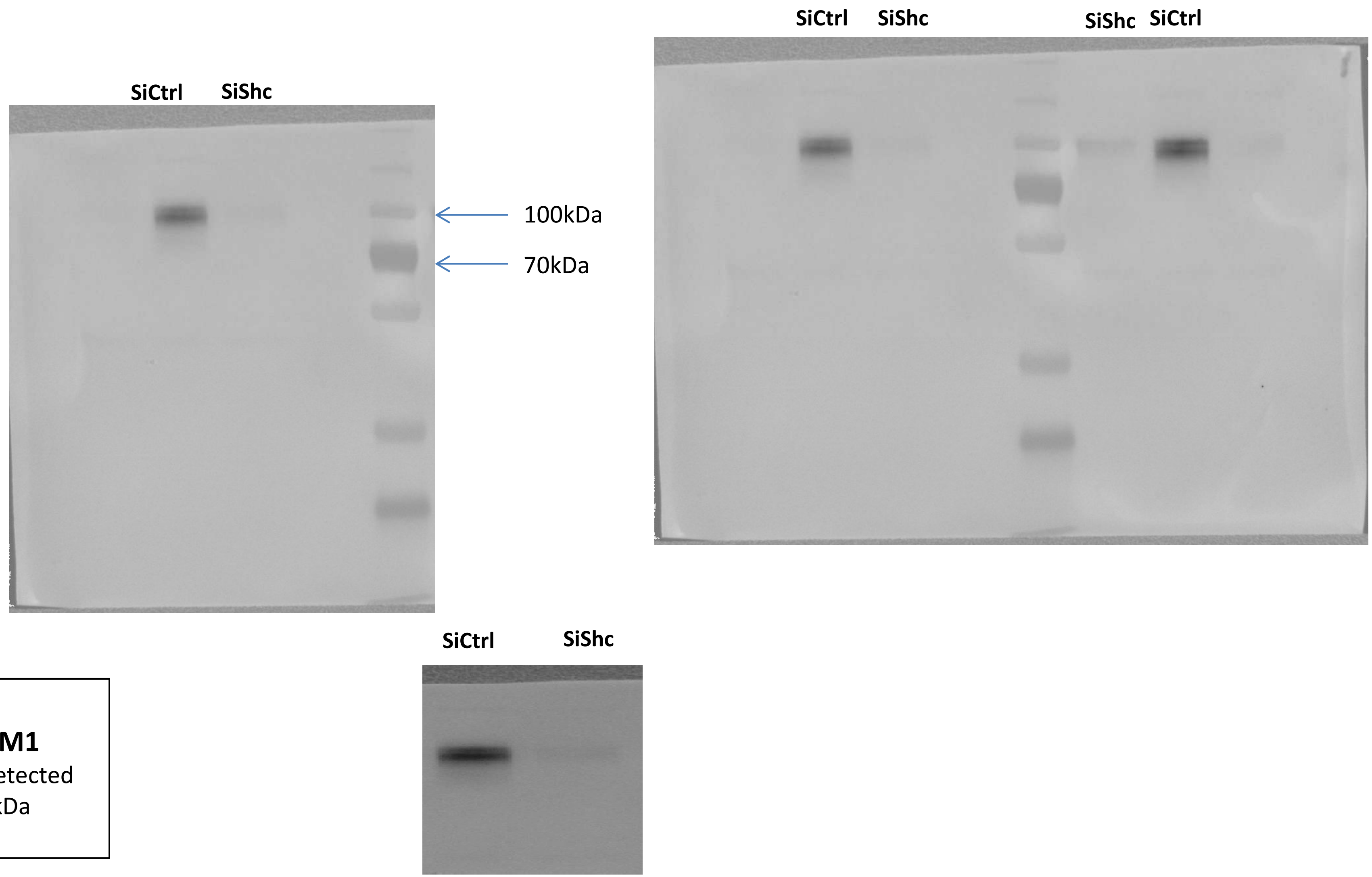

ICAM1
Band detected
89kDa

Figure 2D

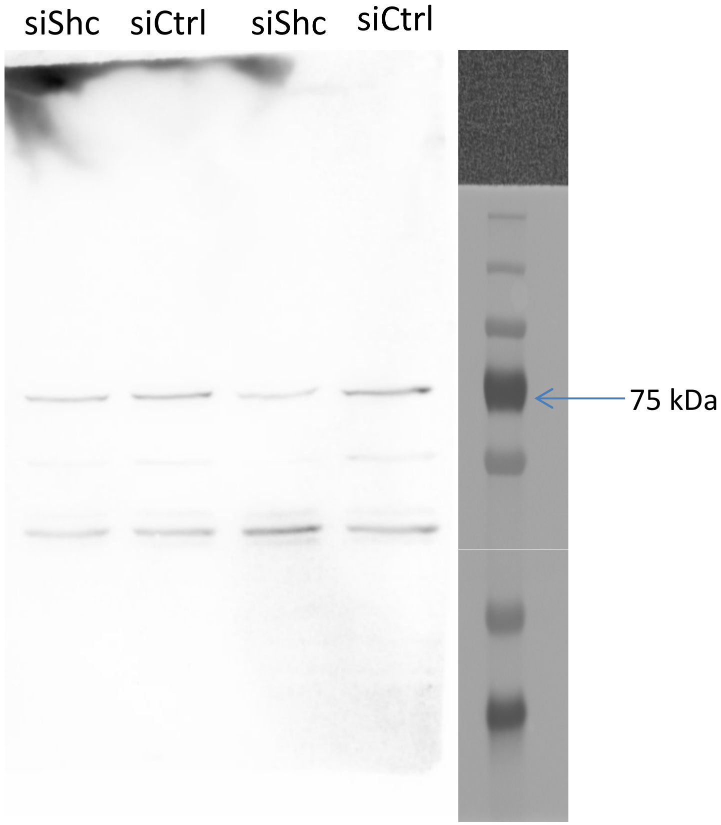

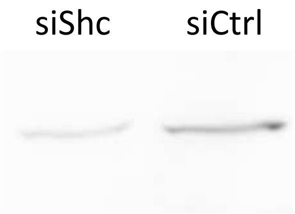

VCAM1

Band detected
81 kDa

Figure 2D

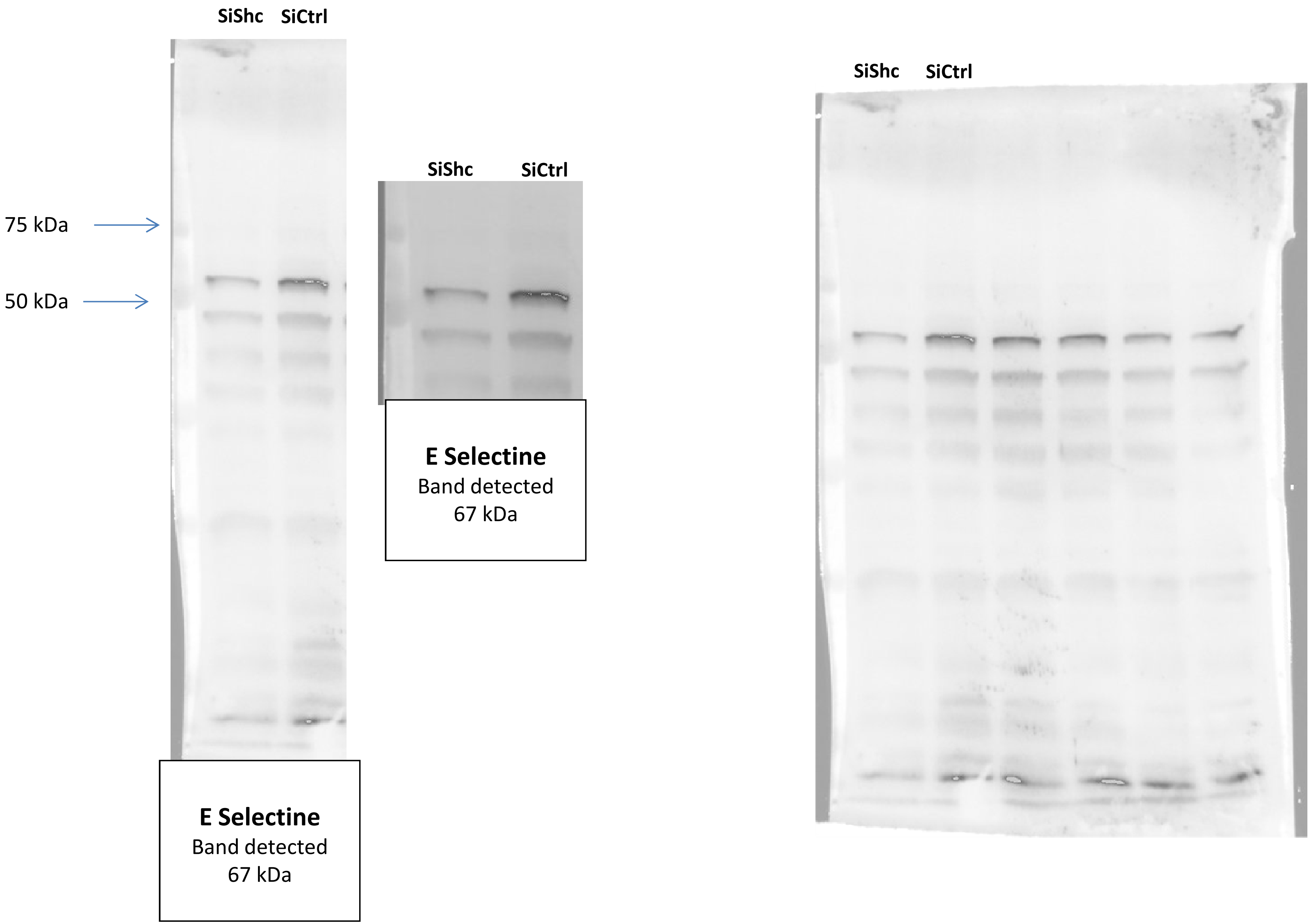
SiShc
SiCtrl
75 kDa
50 kDa
E Selectine
Band detected
67 kDa
SiShc
SiCtrl
E Selectine
Band detected
67 kDa
SiShc
SiCtrl

Figure 2D

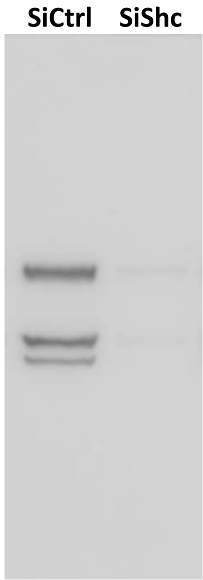

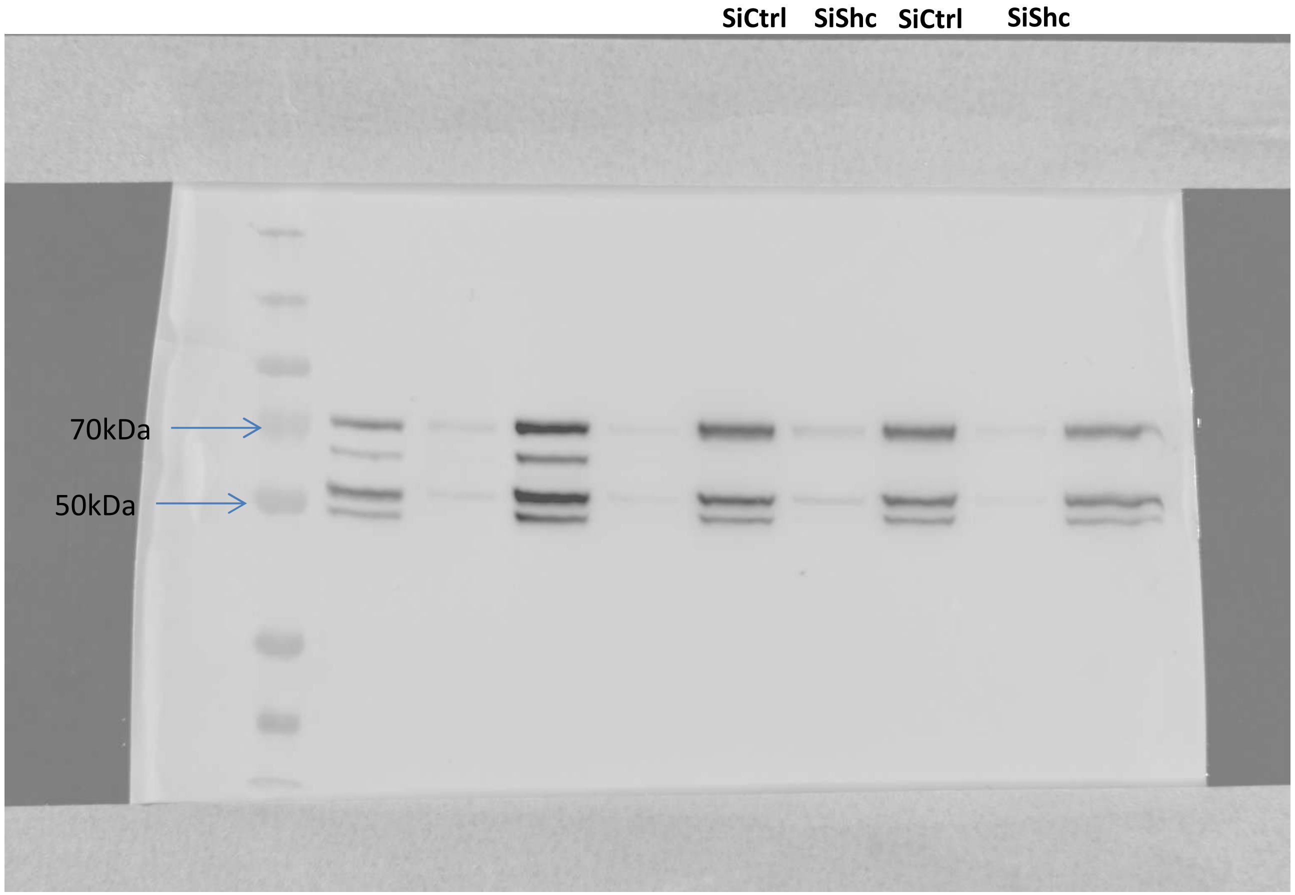

ShcA
66, 52, 46 kDa

Figure 2D

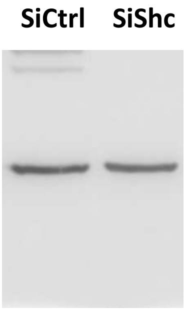

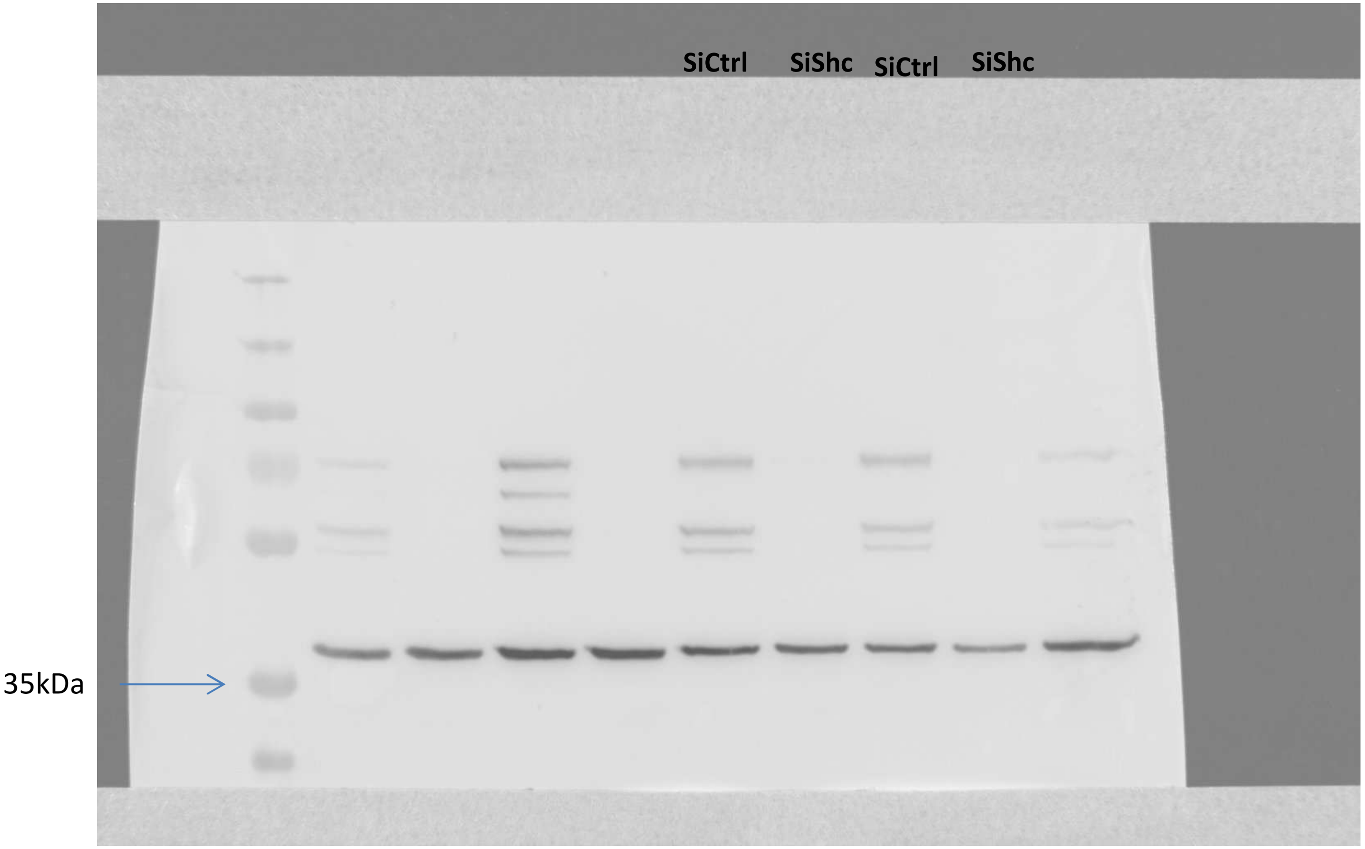

GAPDH
Band detected
39 kDa

ShcA bands re-appear when revealing GAPDH

Figure 3C

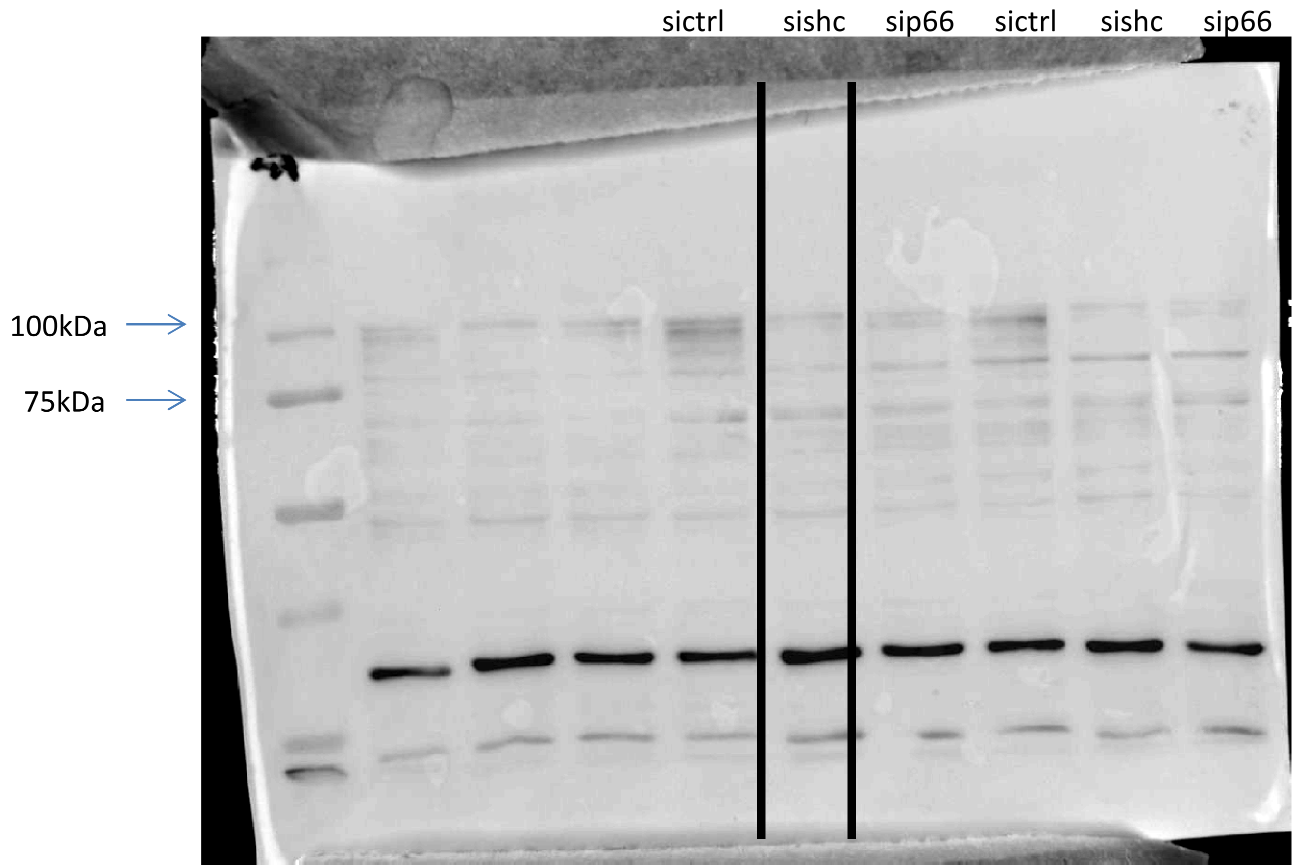

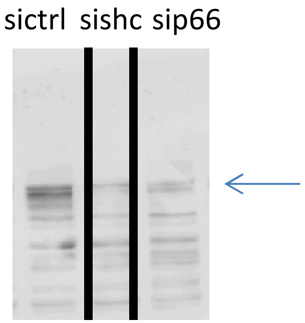

ICAM1
89kDa

Figure 3C

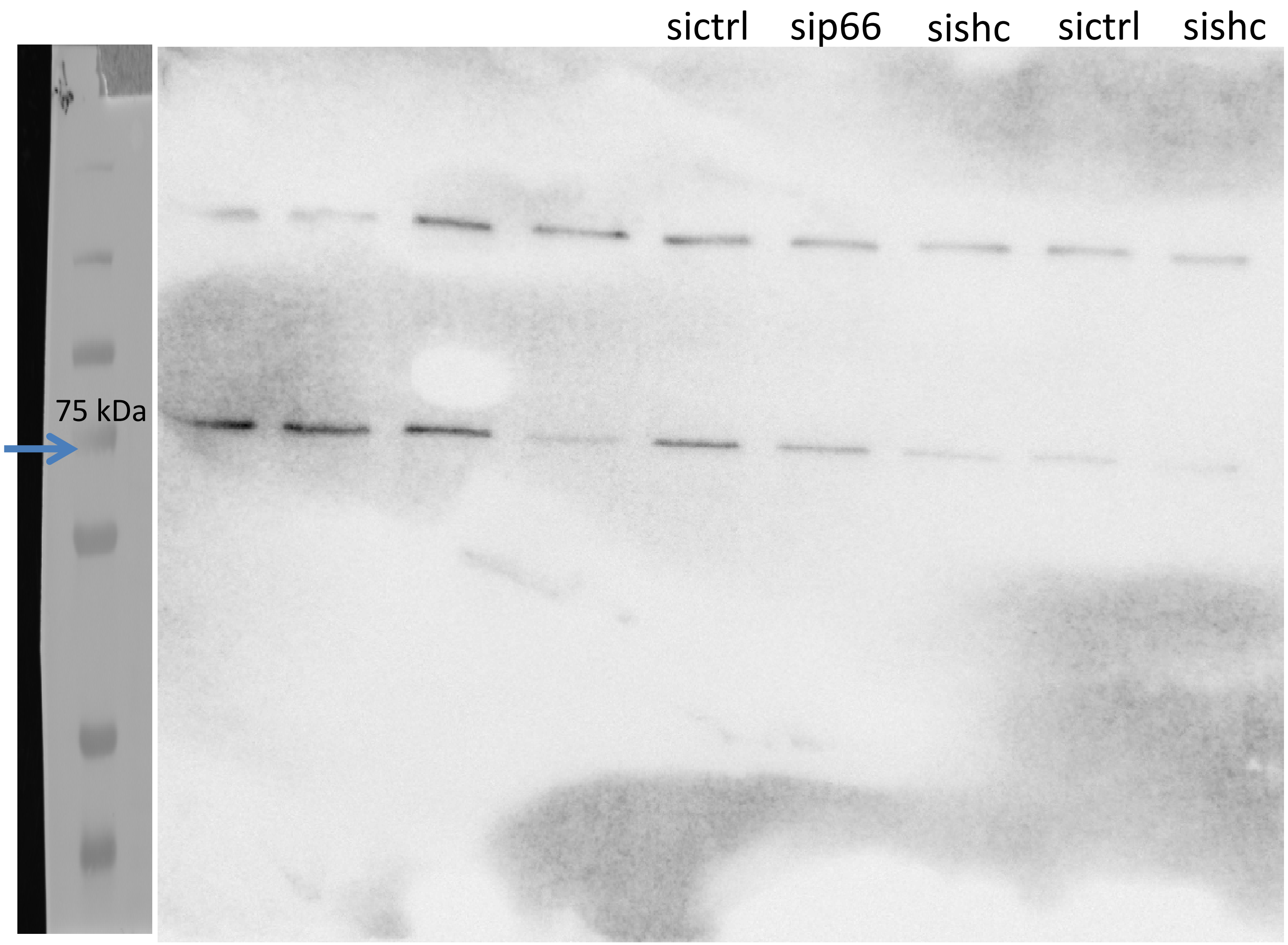

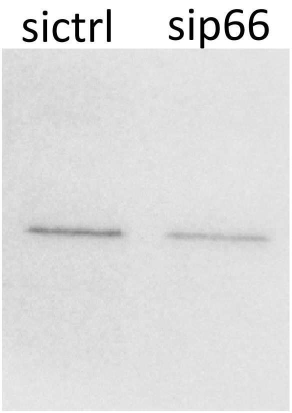

VCAM1

81 kDa

Figure 3C

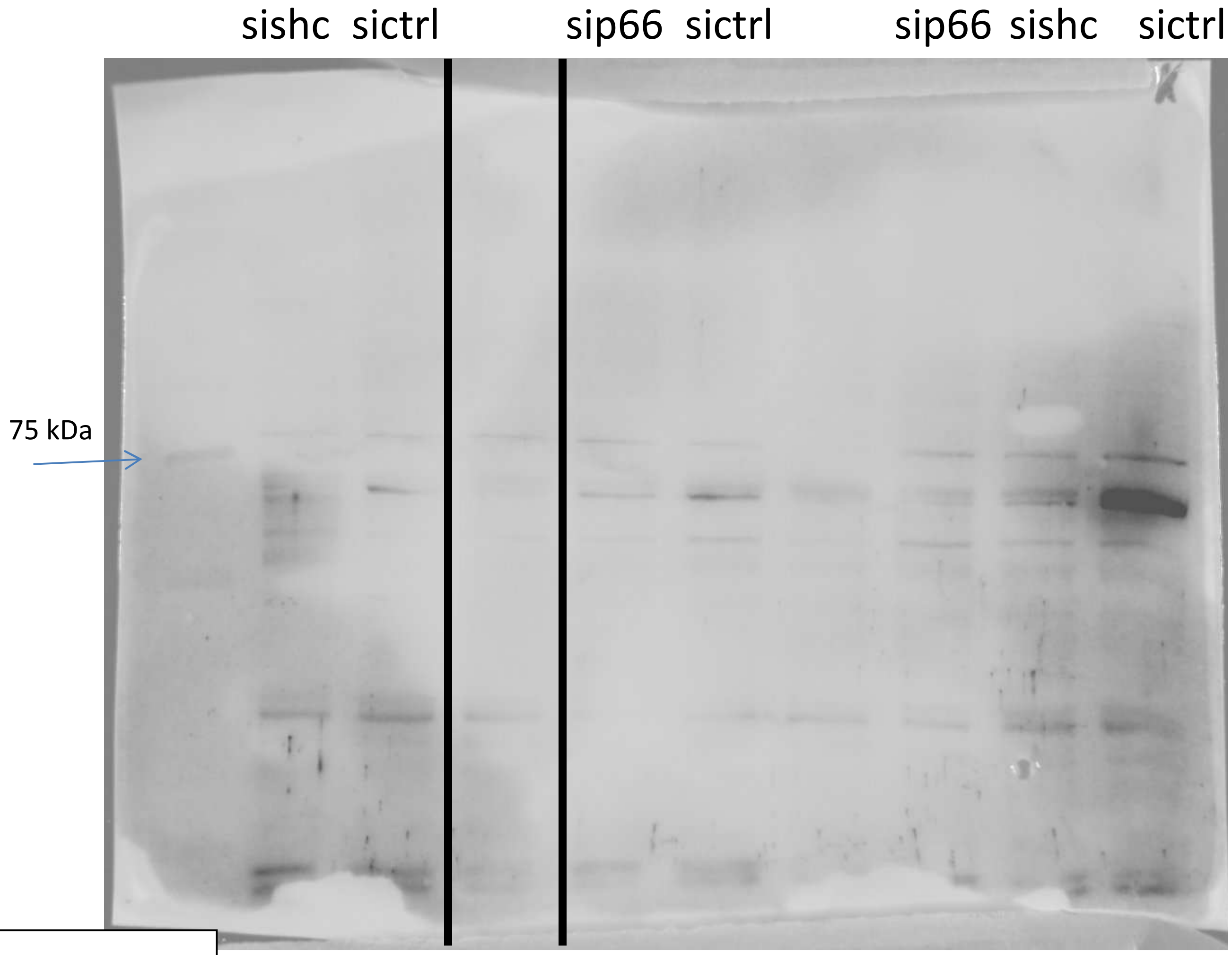

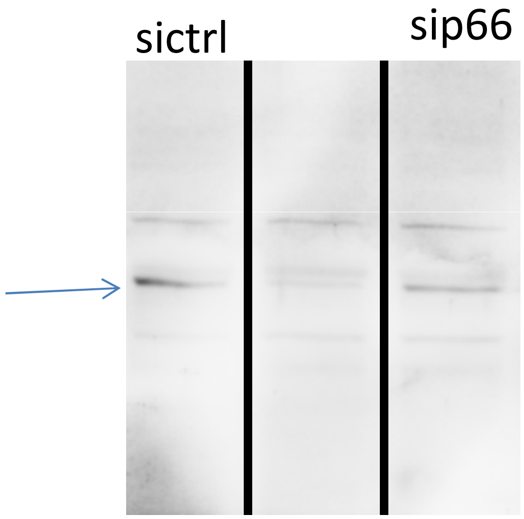

E Selectine
67 kDa

Figure 3C

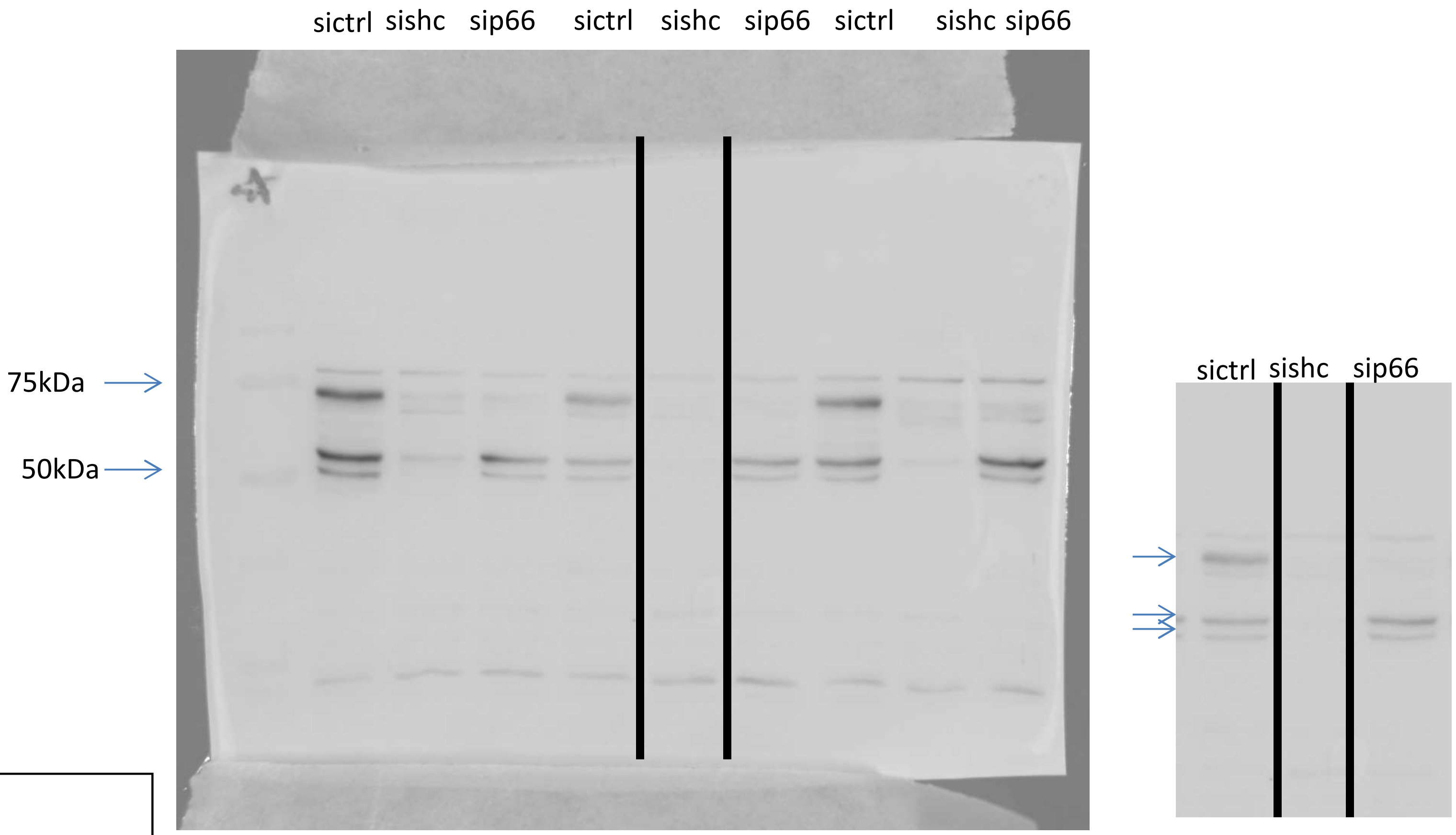

ShcA
66, 52, 46 kDa

Figure 3C

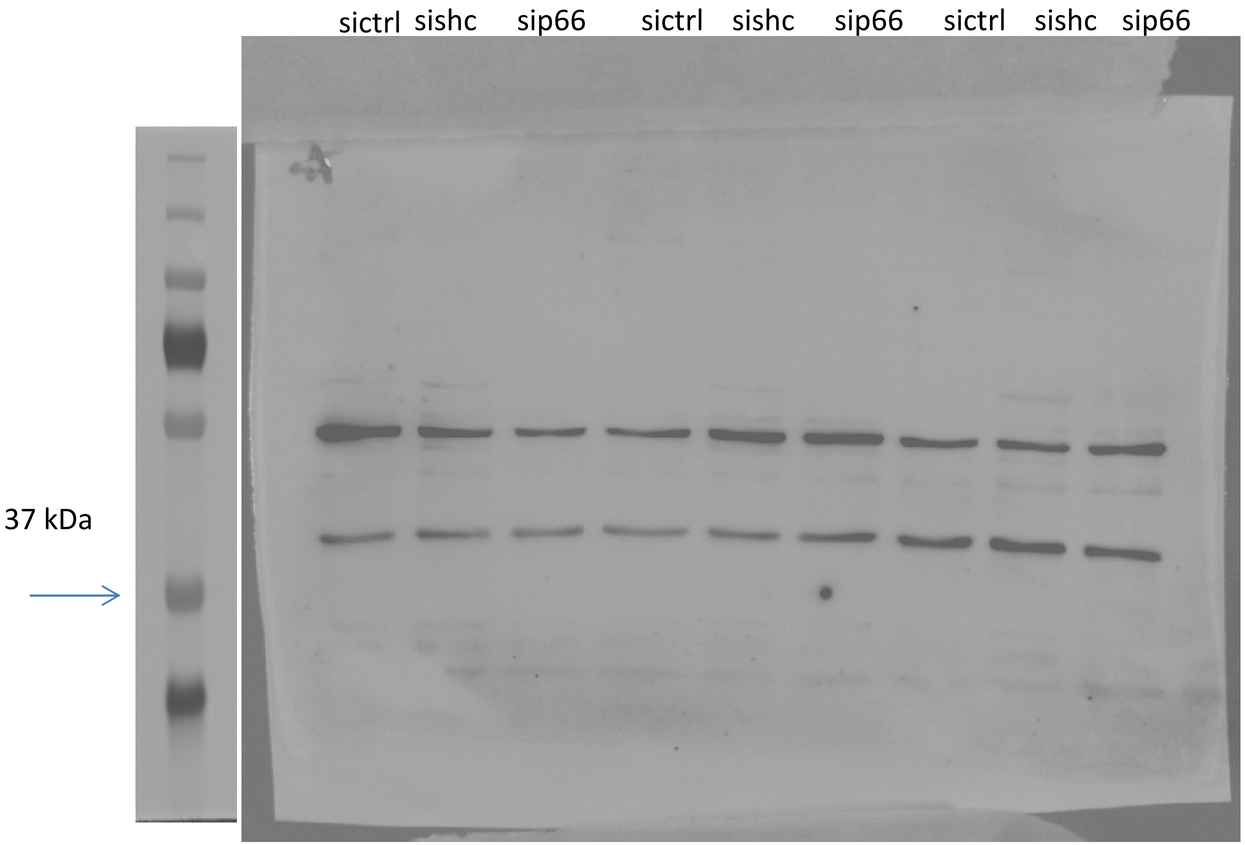

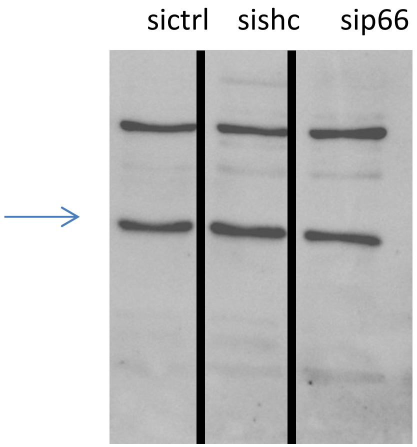

GAPDH
Band detected
39 kDa

ShcA bands re-appear when revealing GAPDH

Figure 4A

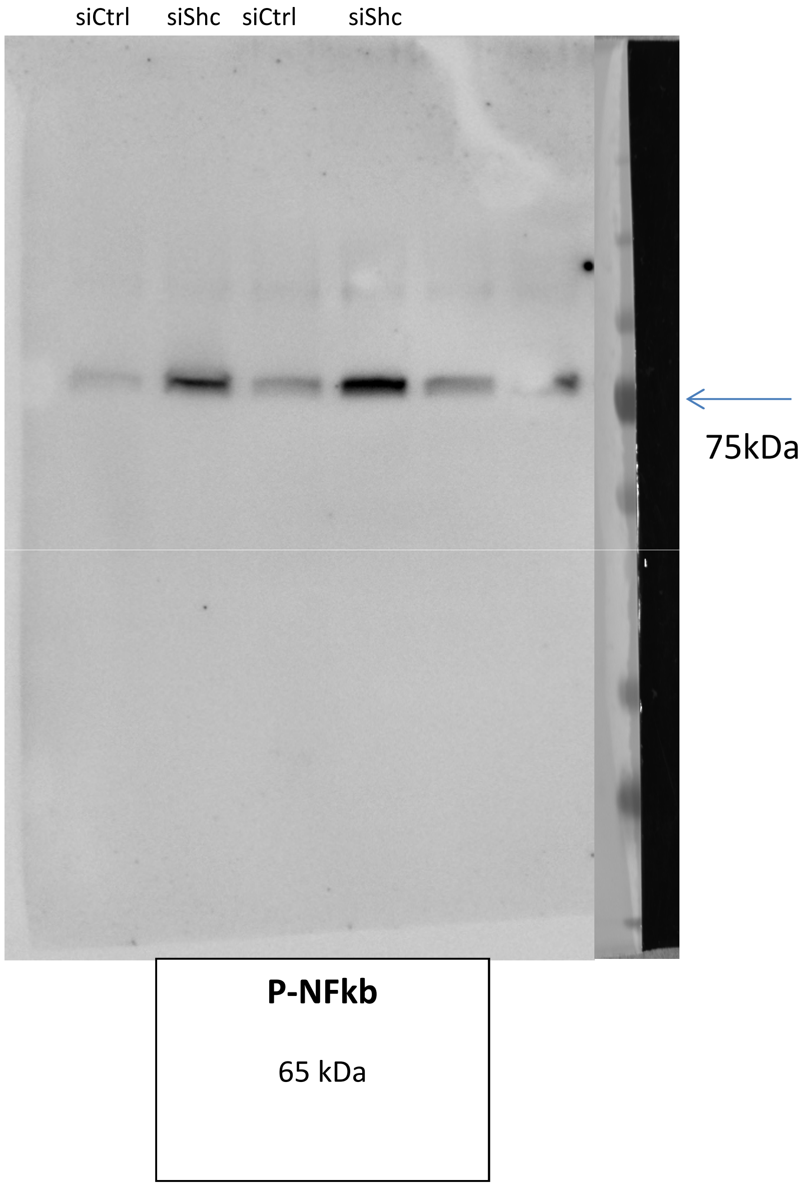

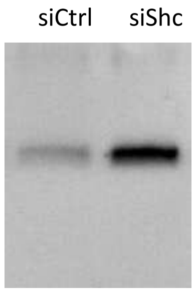

P-NFkb

65 kDa

Figure 4A

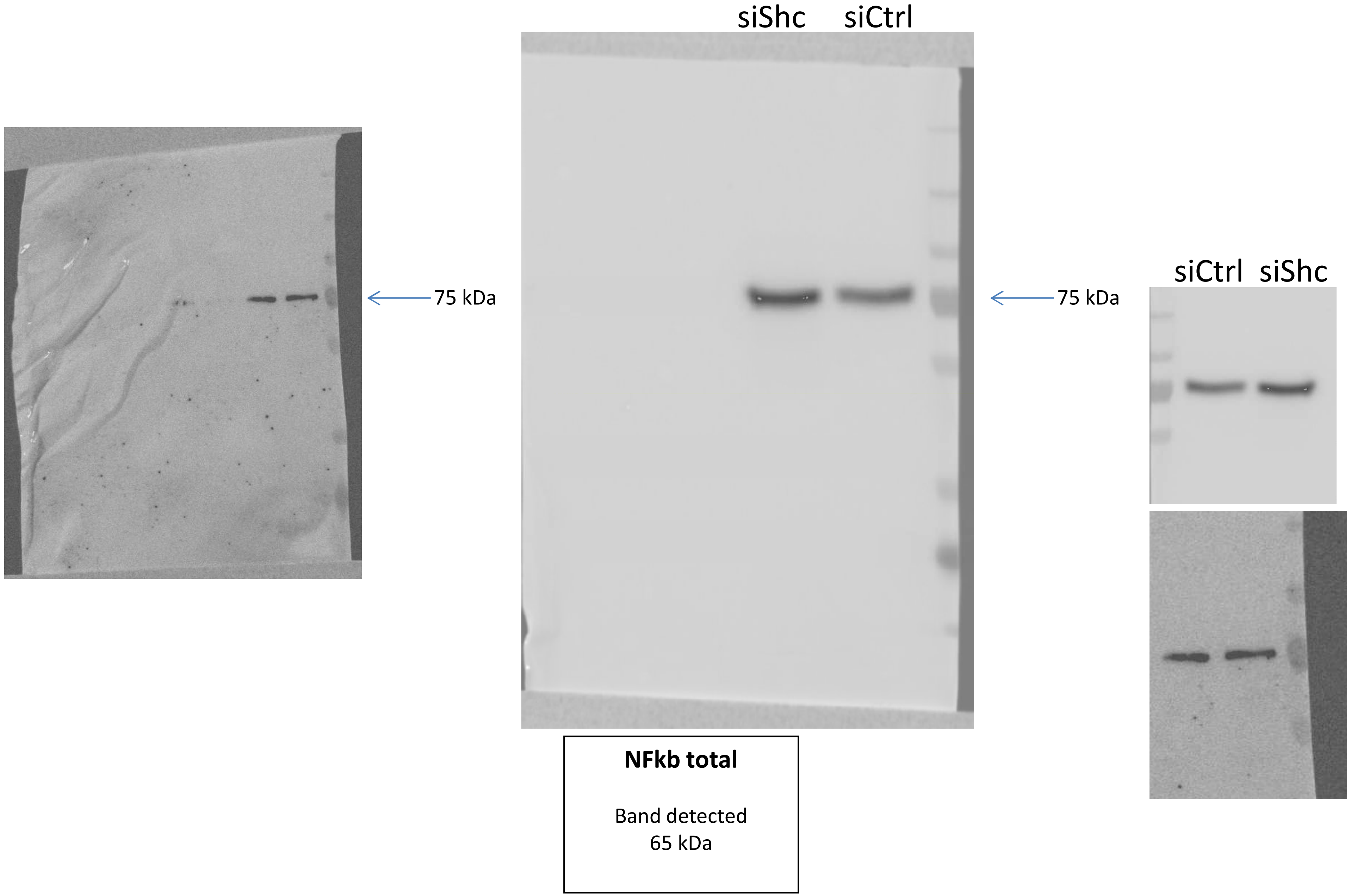

75 kDa
siShc
siCtrl
75 kDa
siCtrl
siShc
NFkb total
Band detected
65 kDa

Figure 4A

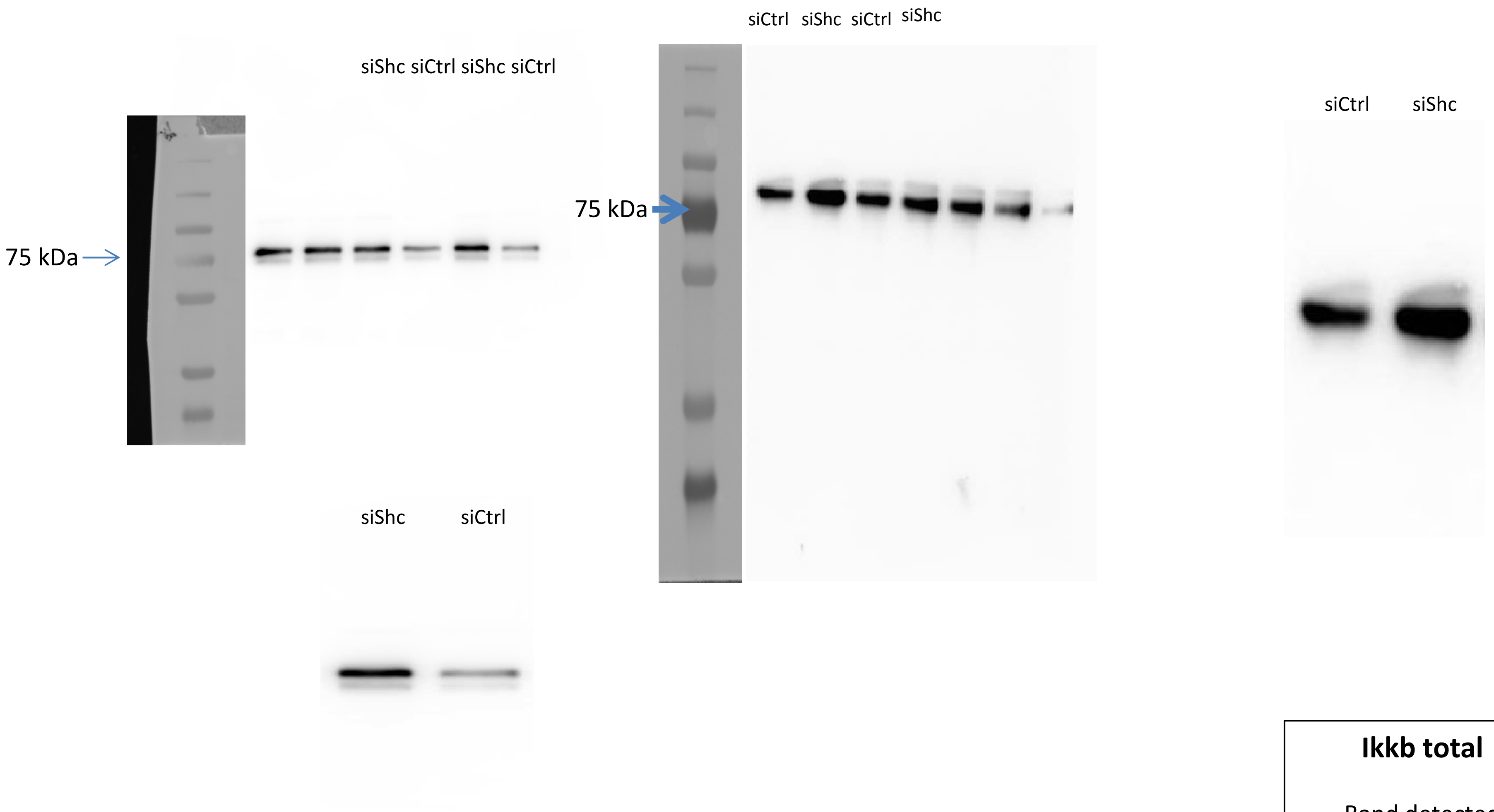

Ikkb total

Band detected
85 kDa

Figure 4A

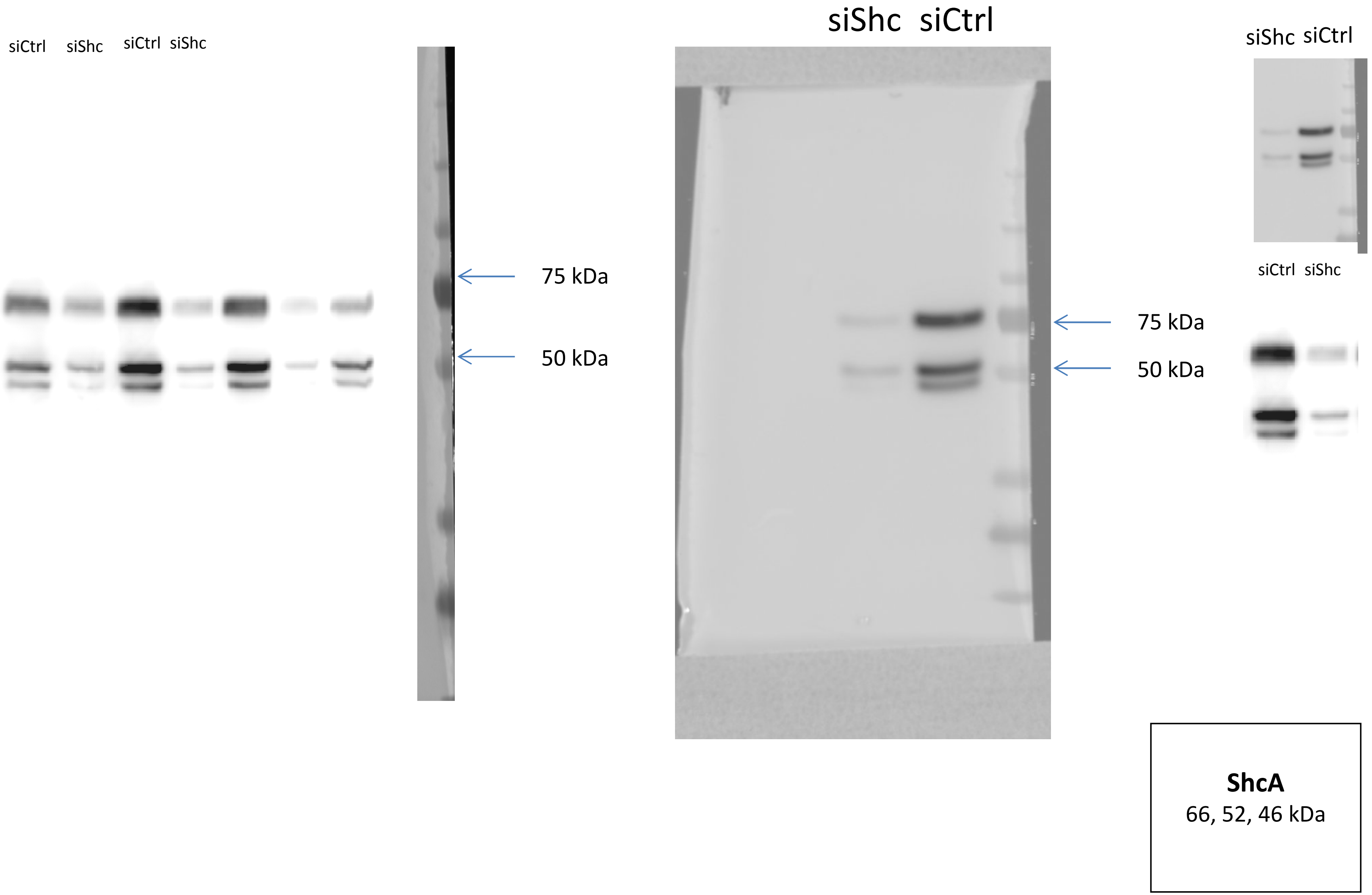

siCtrl siShc siCtrl siShc
75 kDa
50 kDa
siShc siCtrl
75 kDa
50 kDa
siShc siCtrl
siCtrl siShc
ShcA
66, 52, 46 kDa

Figure 4A

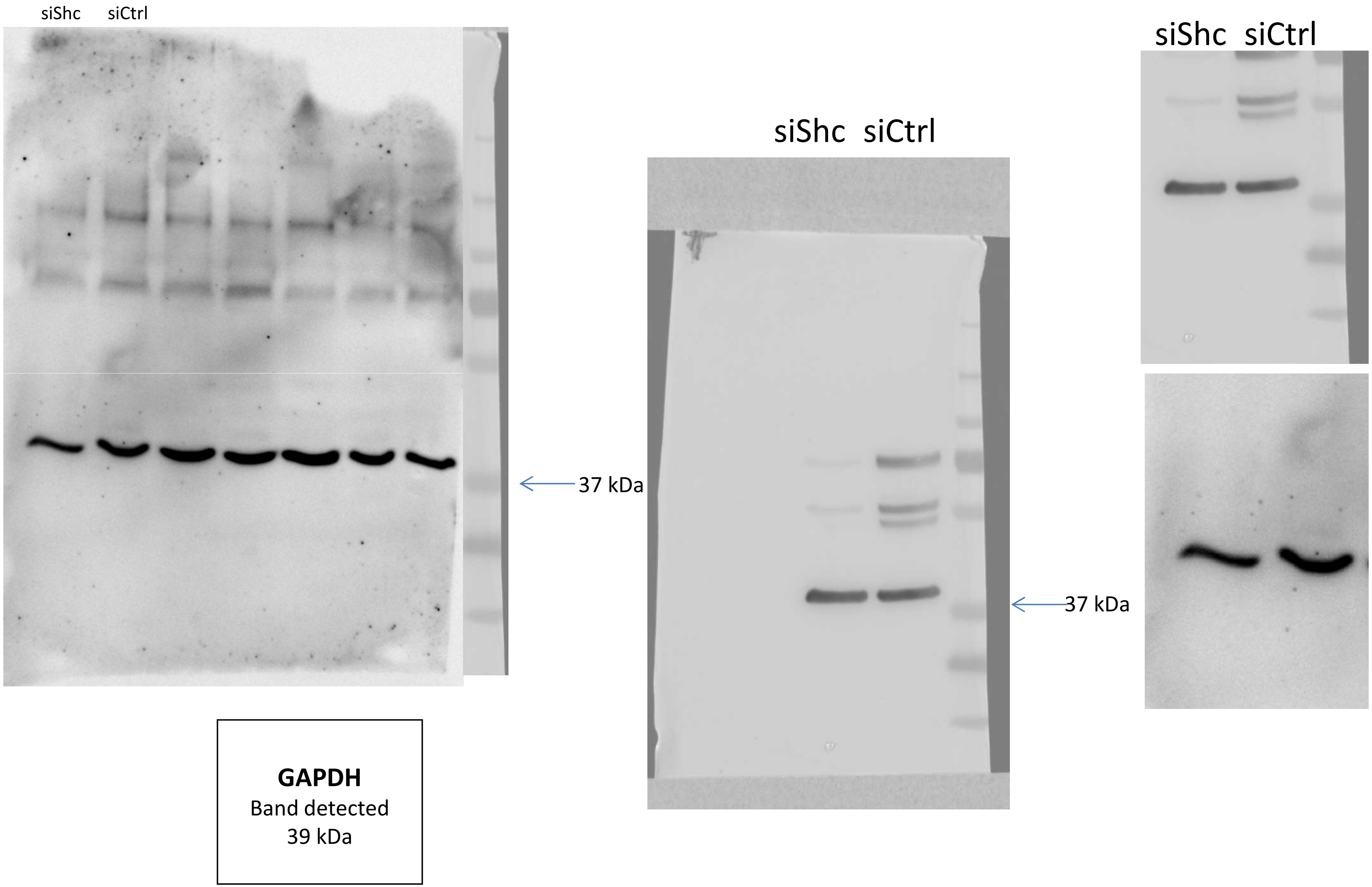

siShc siCtrl
37 kDa
siShc siCtrl
37 kDa
siShc siCtrl
GAPDH
Band detected
39 kDa

Figure 4D

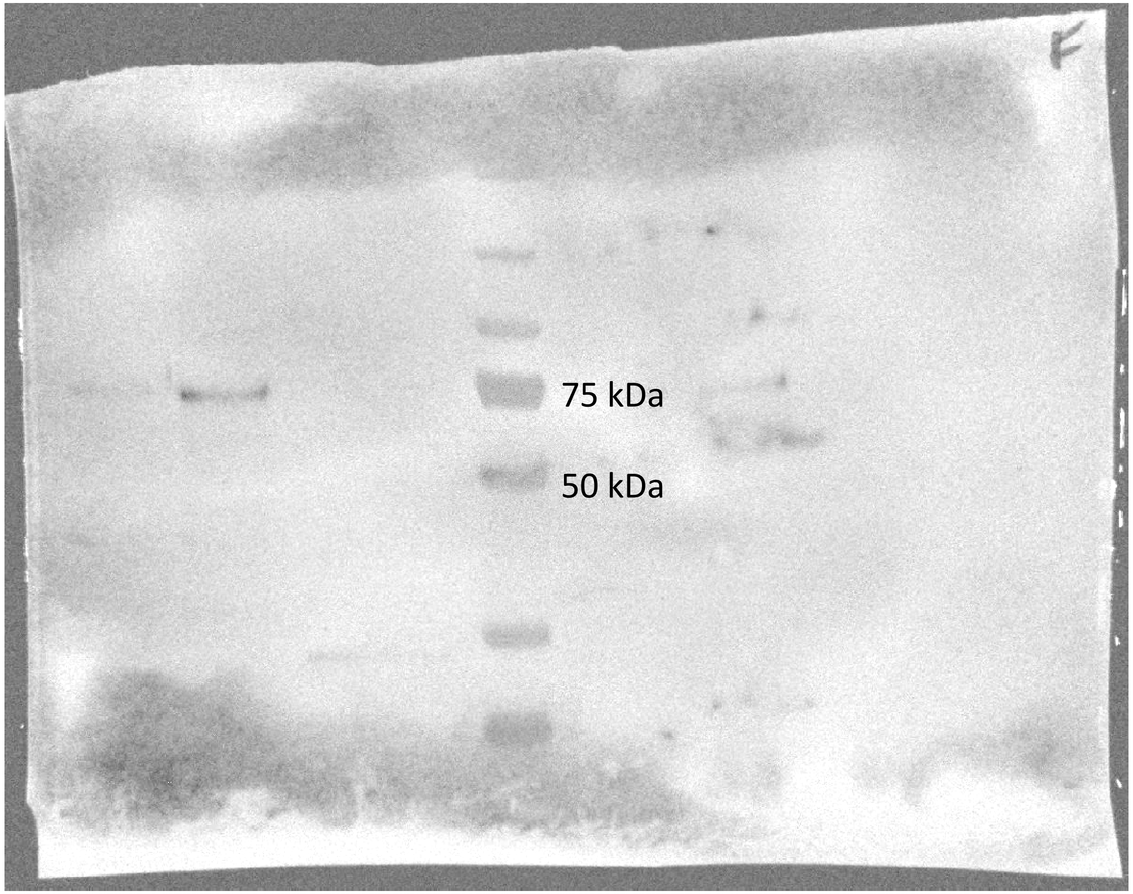

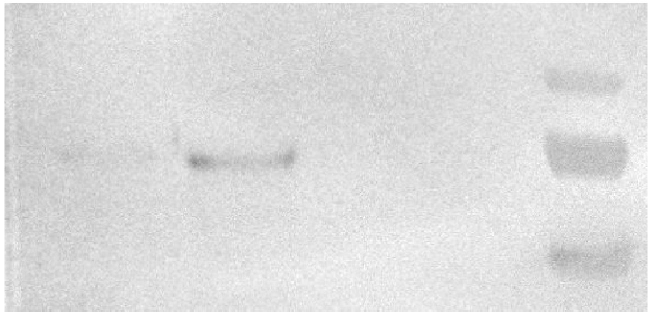

P-NFkb
Cell Fractionation
65 kDa

Figure 4D

siCtrl Nucleus | siShcA Nucleus | siCtrl Cytosol | siShcA Cytosol | siCtrl Nucleus | siShcA Nucleus | siCtrl Cytosol | siShcA Cytosol

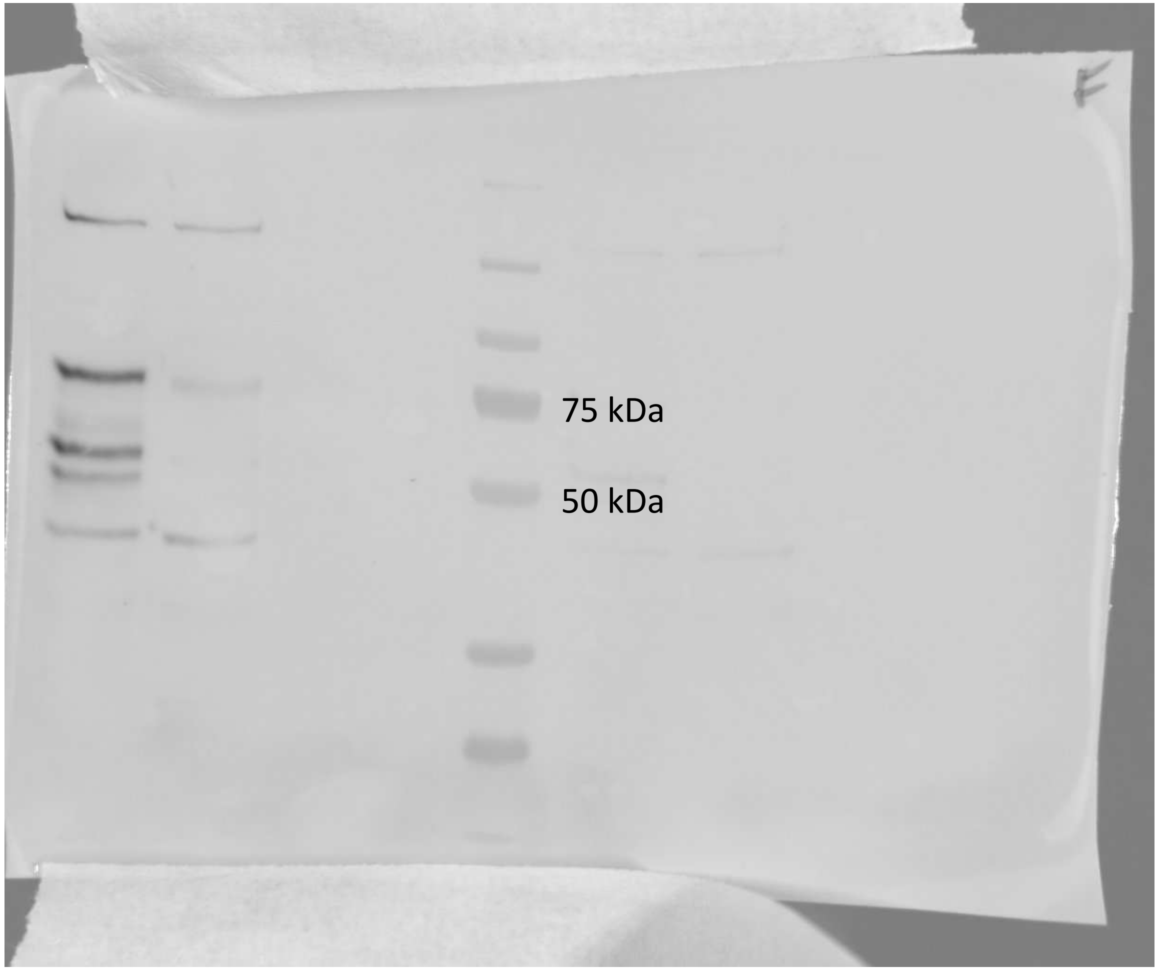

siCtrl Nucleus | siShcA Nucleus

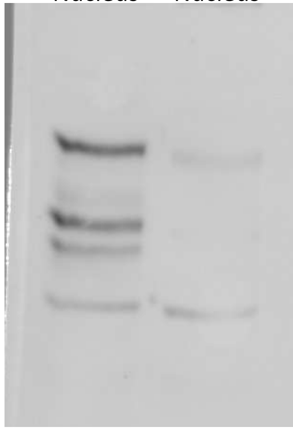

ShcA

66, 52, 46 kDa

Figure 4D

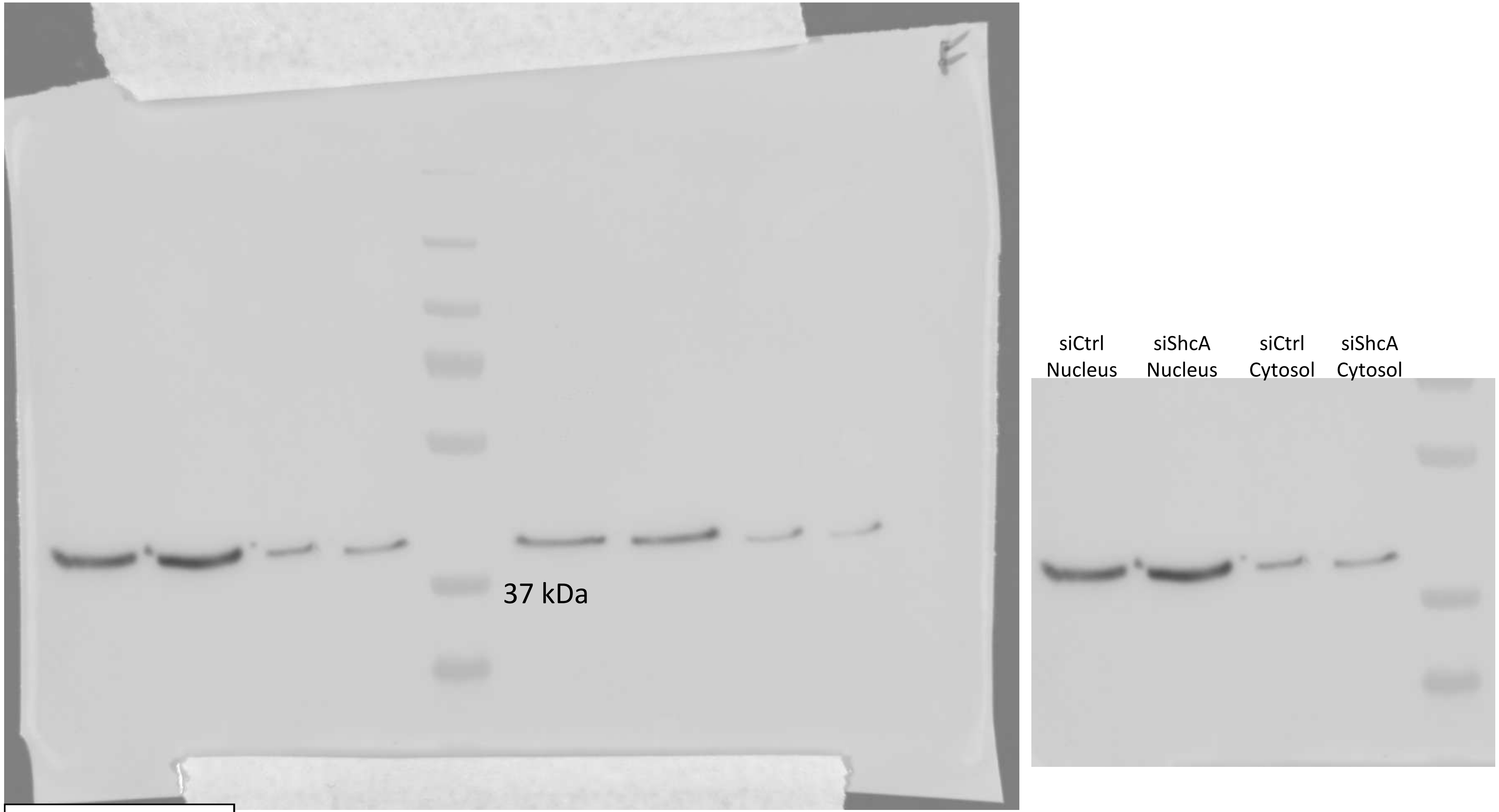

GAPDH
Band detected
39 kDa

Figure 5B

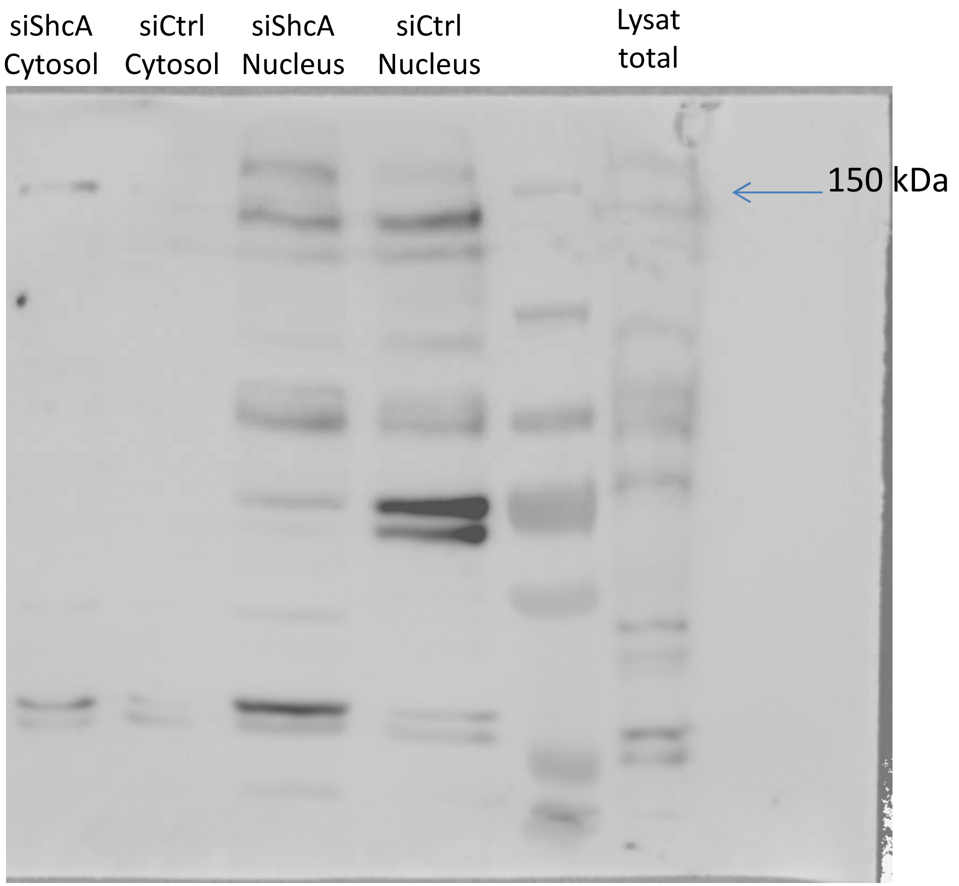

ZEB1
Cell Fractionation
200 kDa

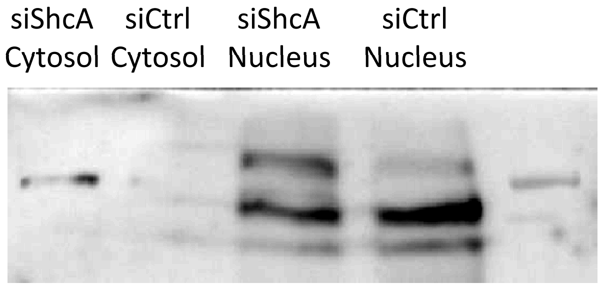

The contrast adjustment to the original blot (shown here) and to the published image may not be the same.

Figure 5B

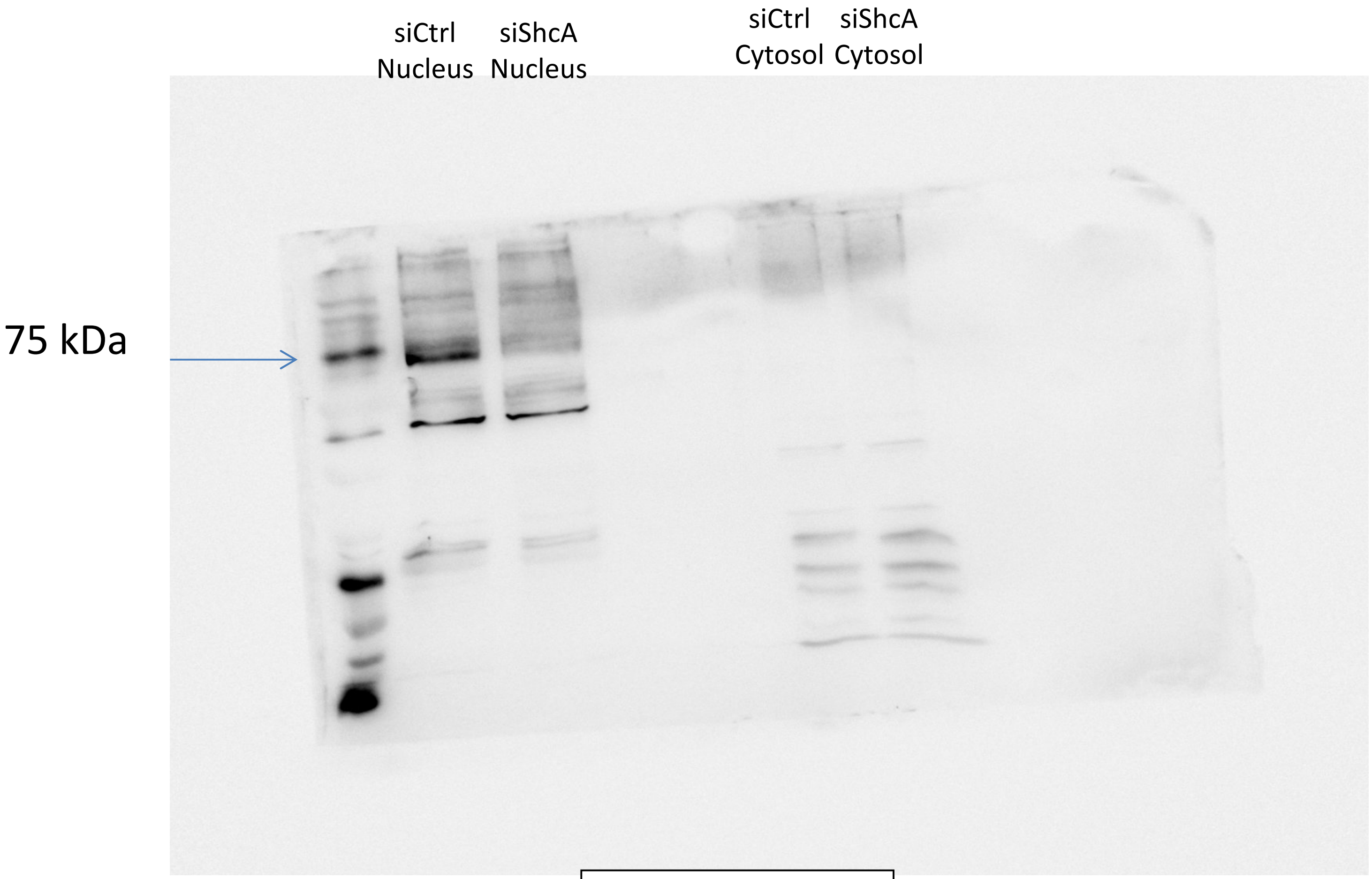

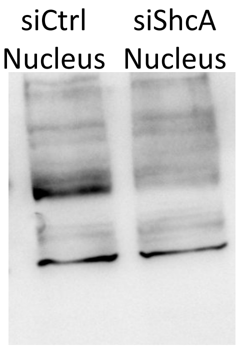

YAP1
Cell Fractionation
70 kDa

Figure 5B

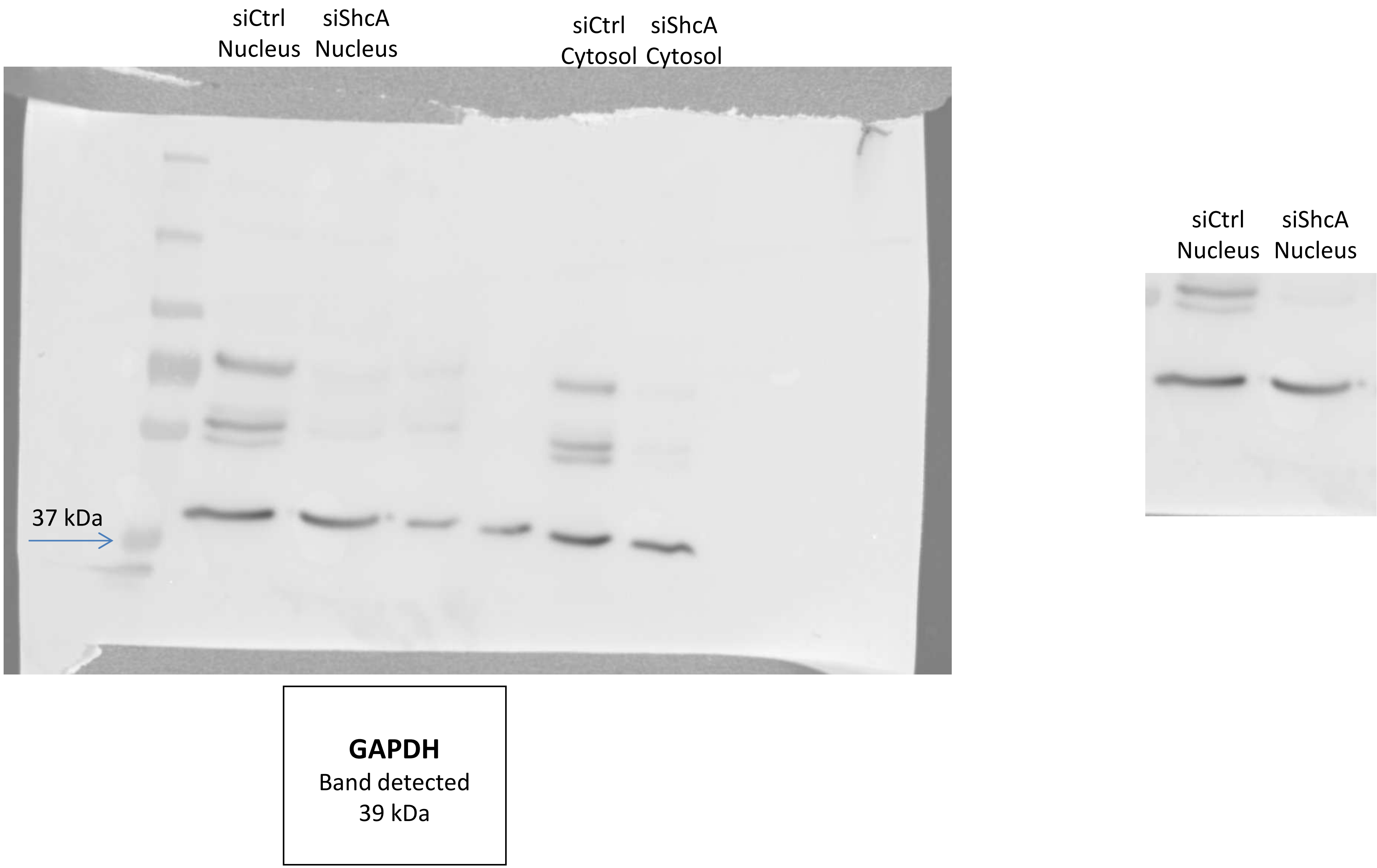
siCtrl Nucleus
siShcA Nucleus
siCtrl Cytosol
siShcA Cytosol
37 kDa
siCtrl Nucleus
siShcA Nucleus
GAPDH
Band detected
39 kDa

Figure 5C

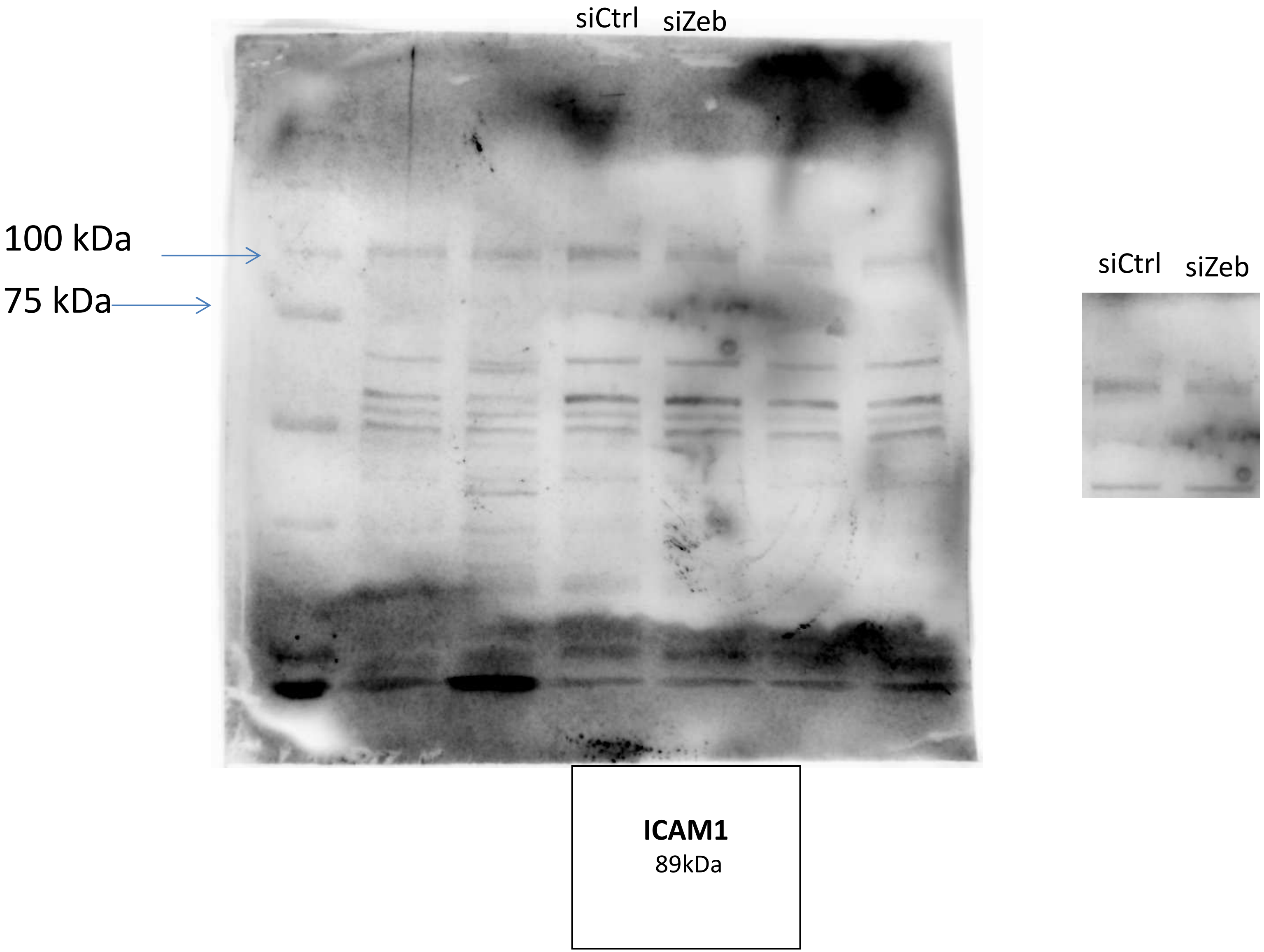

Figure 5C

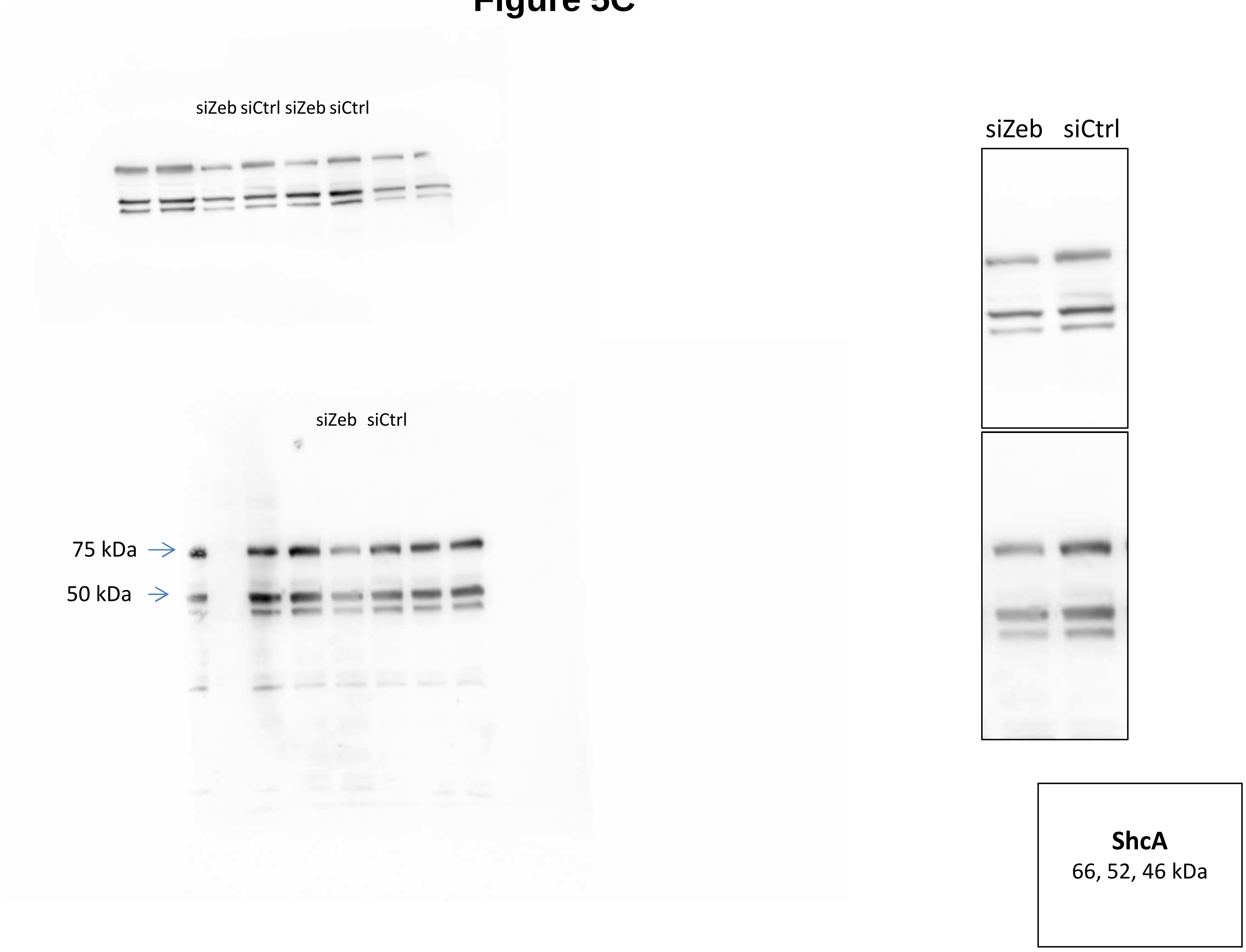
siZeb siCtrl siZeb siCtrl
siZeb siCtrl
75 kDa
50 kDa
siZeb siCtrl
ShcA
66, 52, 46 kDa

Figure 5C

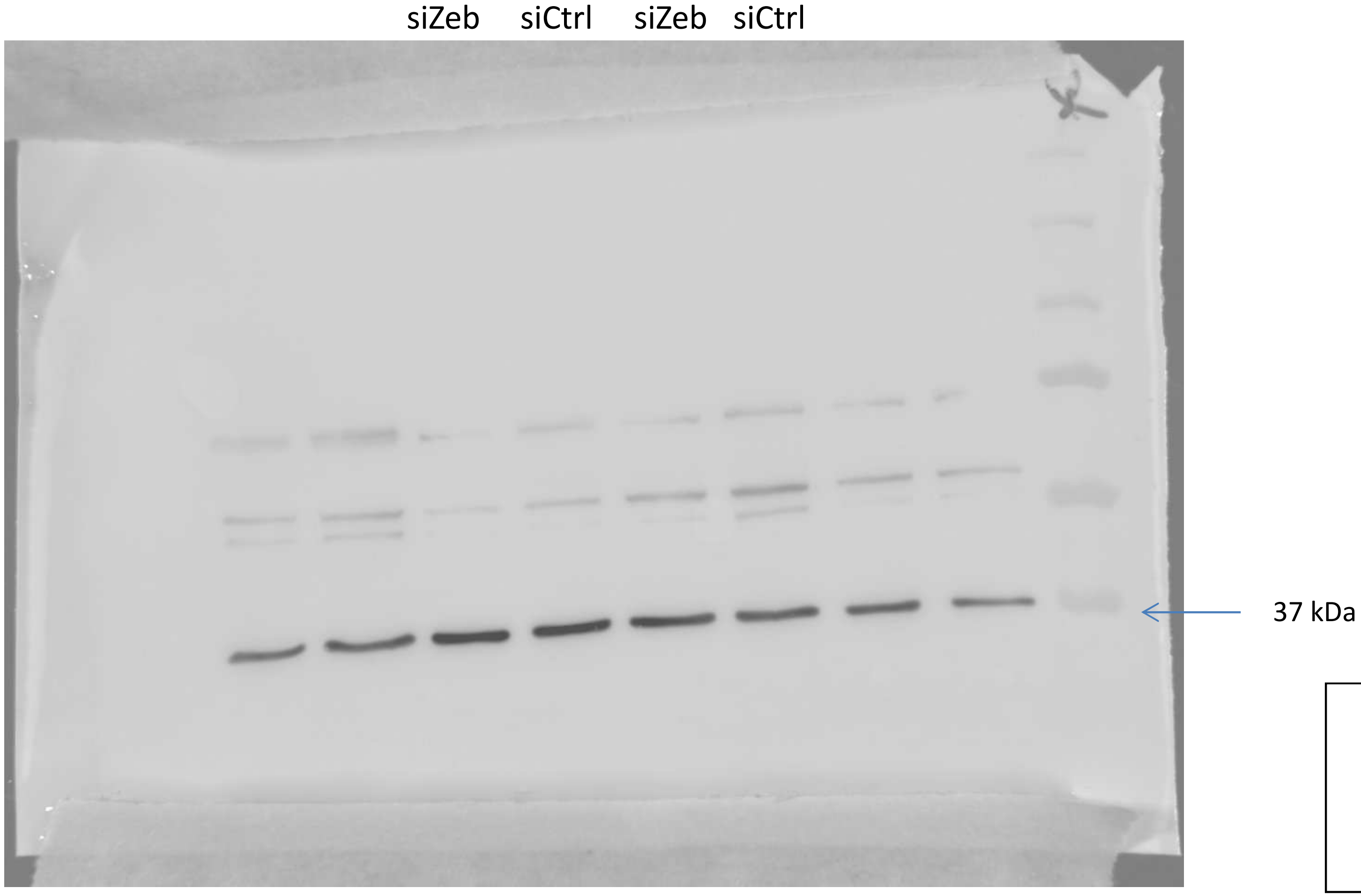

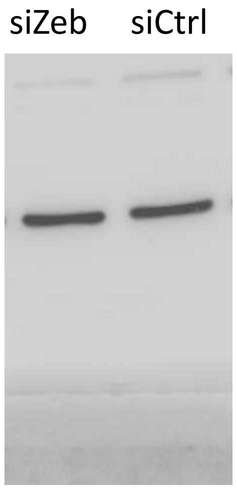

GAPDH
Band detected
39 kDa

Figure 6A

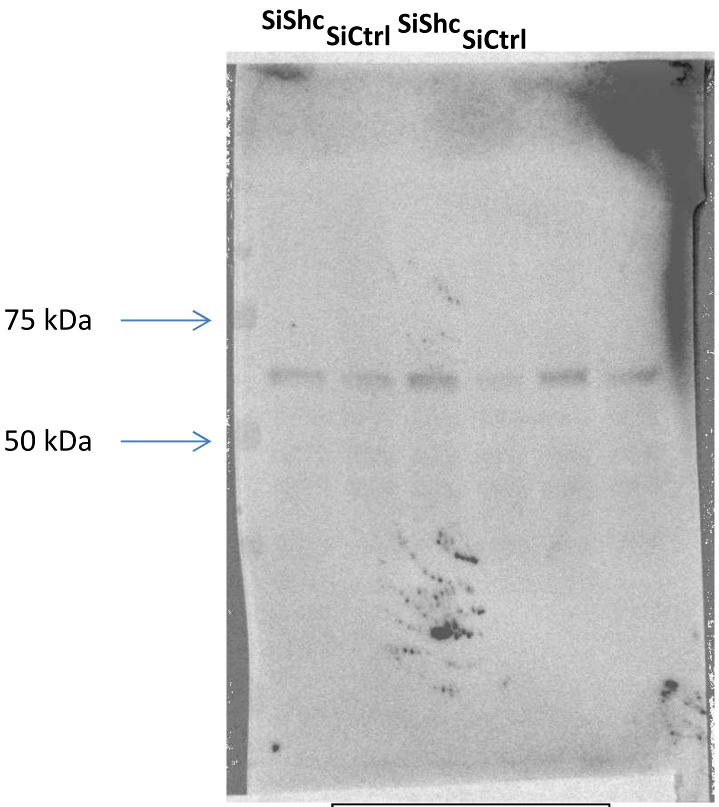

P-Akt
60 kDa

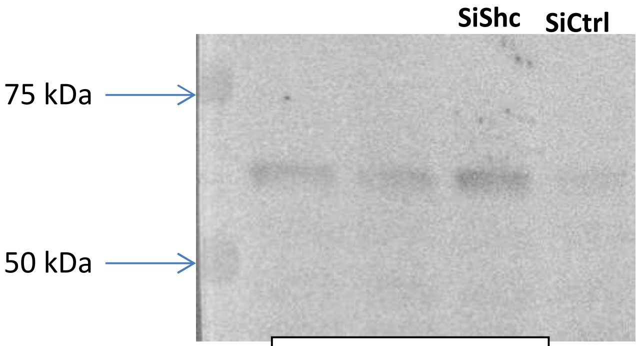

P-Akt
Band detected
60 kDa

Figure 6A

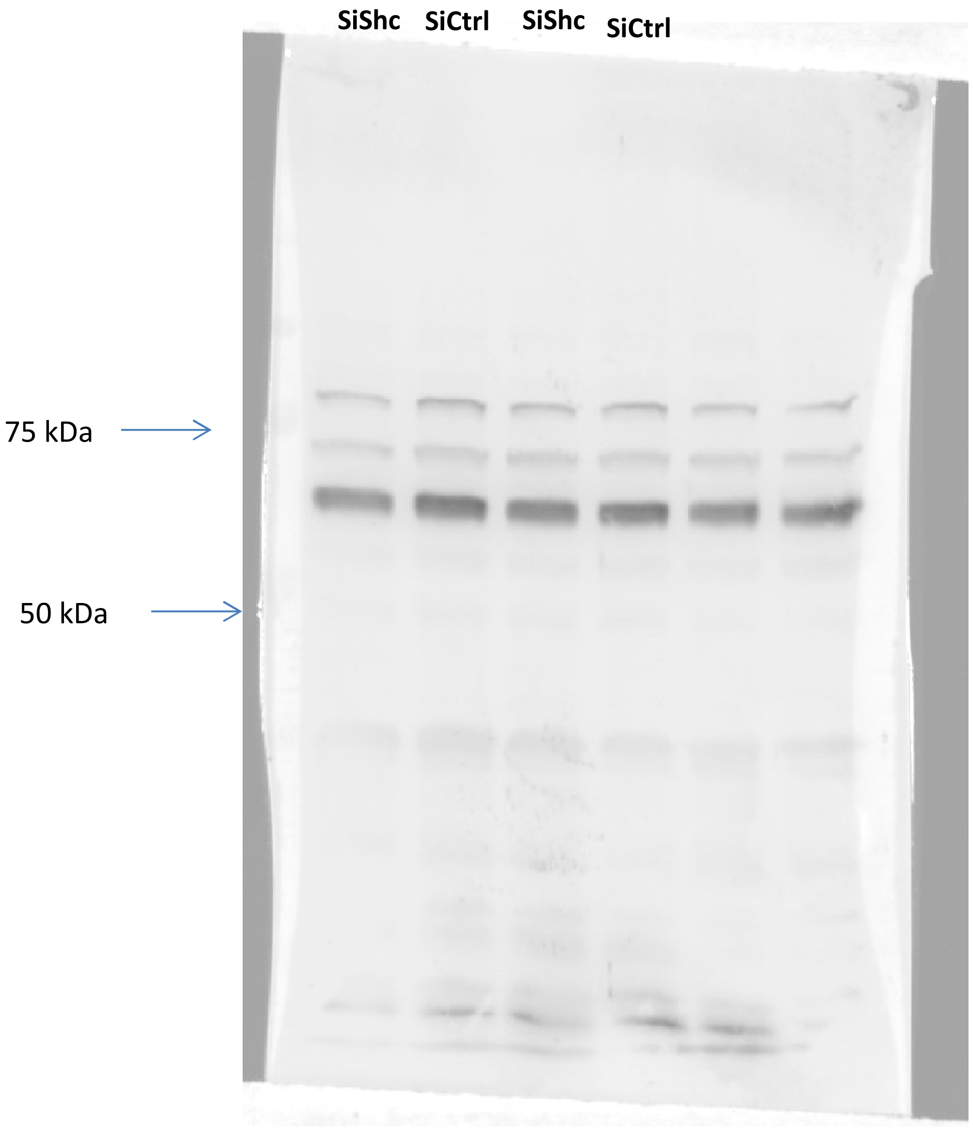

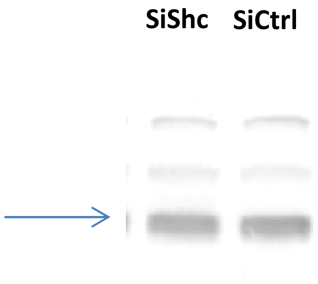

Akt Total
60 kDa

Figure 6A

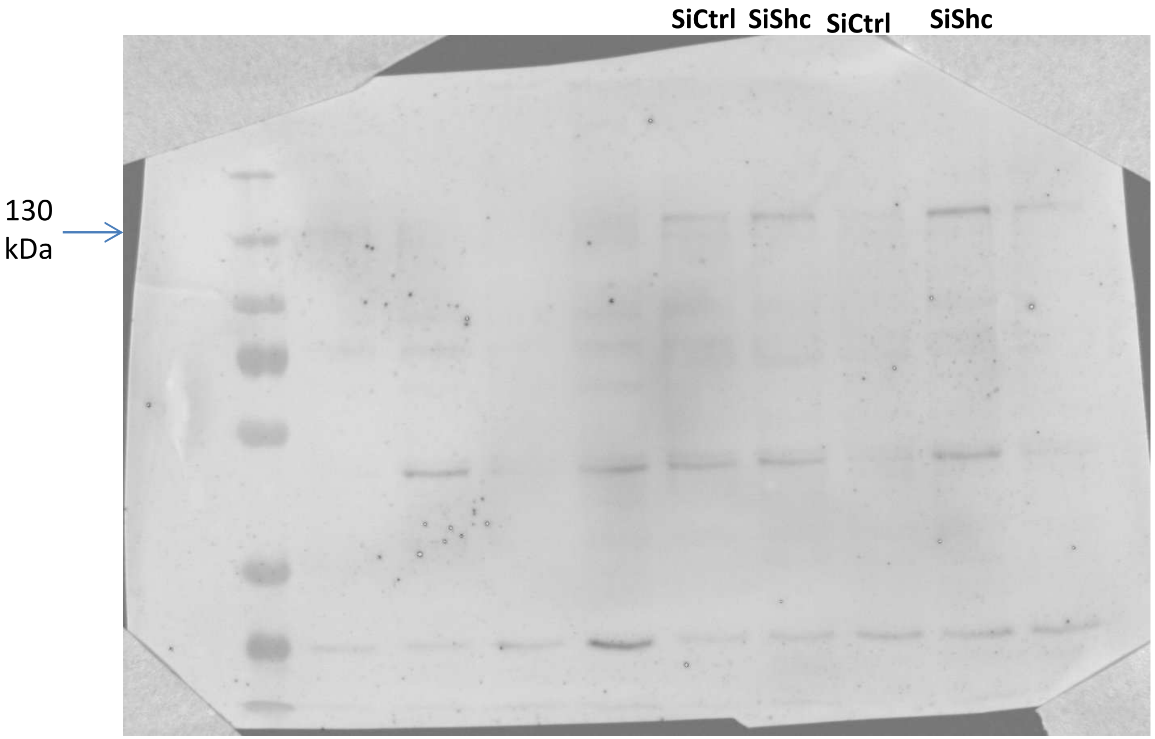

P-enos

140 kDa

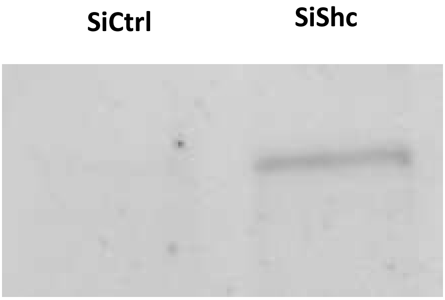

Figure 6A

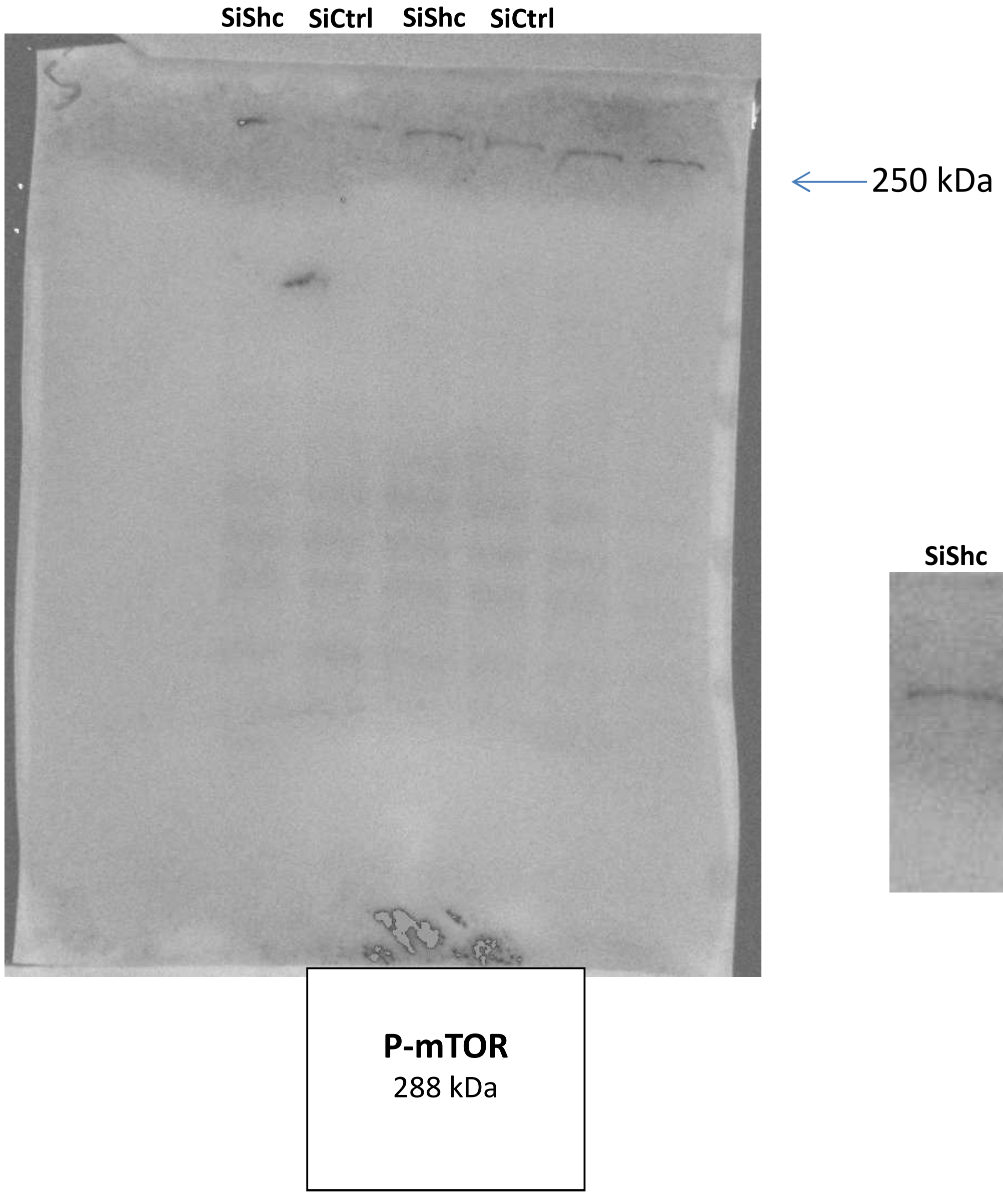

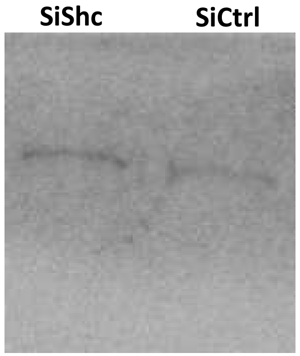

P-mTOR
288 kDa

Figure 6A

SiShc SiCtrl SiShc SiCtrl SiShc

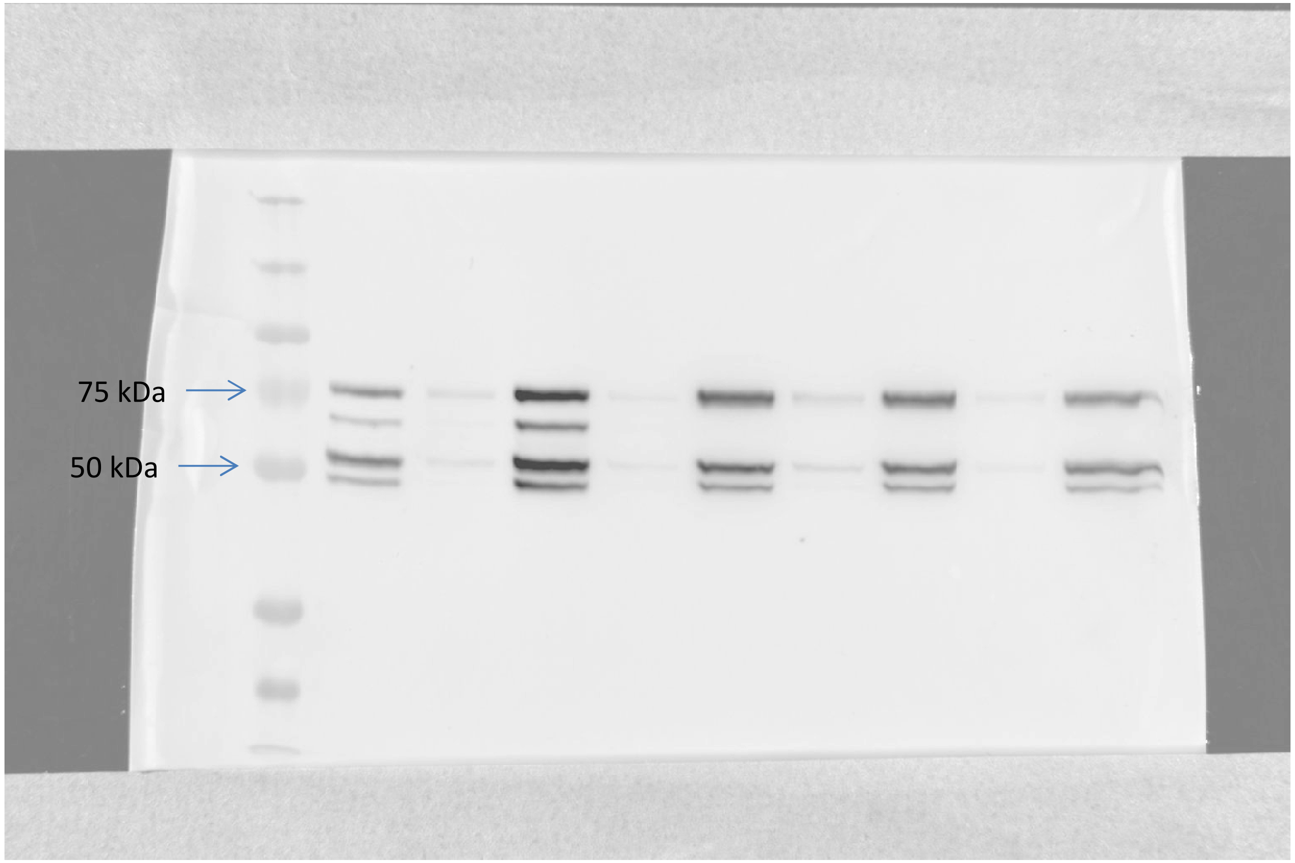

SiCtrl SiShc

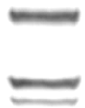

ShcA
66, 52, 46 kDa

Several blots were revealed prior to ShcA.
Residual signal still appears from previous blots.

Figure 6A

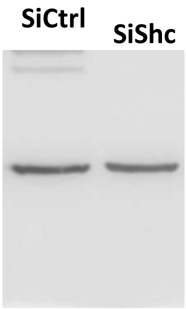

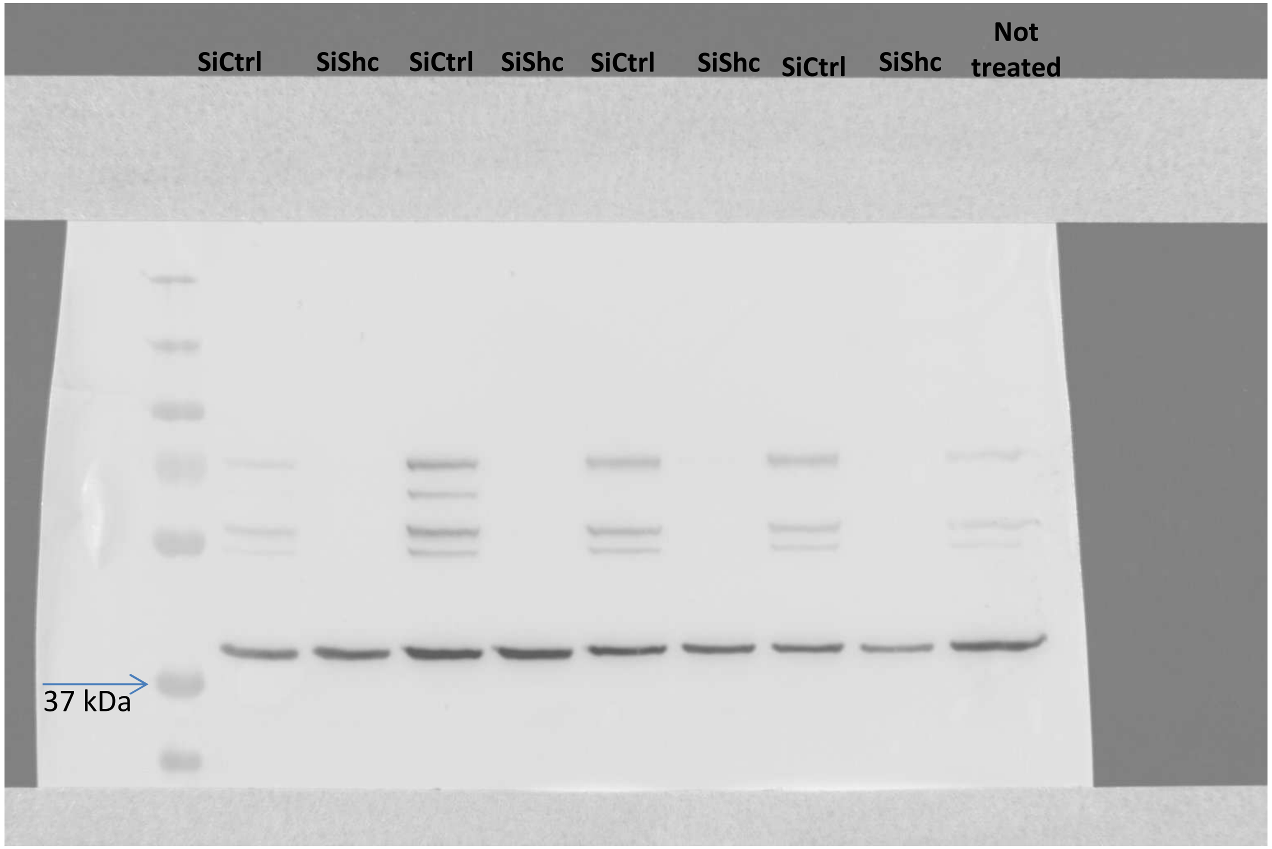

GAPDH
Band detected
39 kDa
